# A Color Image Encryption Using a 4D Variable-Order Fractional Hyperchaotic System and Chess-Gameplay-Inspired Dynamic Mechanism

**DOI:** 10.3390/e28070795

**Published:** 2026-07-13

**Authors:** Xiaomeng Cui, Xiaoqiang Zhang, Jiaqi Ji

**Affiliations:** 1 School of Mathematics and Computer Science, Hebei Minzu Normal University, Chengde 067000, China; xmc@hbun.edu.cn (X.C.); jijiaqi@hbun.edu.cn (J.J.); 2 School of Information and Control Engineering, China University of Mining and Technology, Xuzhou 221116, China

**Keywords:** image encryption, fractional-order system, variable order, hyperchaotic system, coupled permutation–diffusion

## Abstract

With the widespread adoption of digital images in network transmission and storage, the demand for image privacy protection keeps rising. We propose a robust scheme combining a four-dimensional variable-order fractional hyperchaotic system (4D-VOFHS) and a chess-game play-inspired dynamic mechanism. Firstly, we construct 4D-VOFHS, to overcome inherent limitations of constant-order systems: unlike constant-order systems that are vulnerable to deep-learning-based parameter identification attacks, this system introduces time-varying orders and high-dimensional coupling to enrich nonlinear dynamics. Secondly, inspired by the dynamic strategic interactions within chess gameplay, we design a synchronous encryption framework with a tightly coupled permutation–diffusion mechanism. This design not only significantly enhances the nonlinear complexity, confusion and diffusion performance of the algorithm, but also enables parallel synchronous processing to improve computational throughput. Finally, we propose a block-based collaborative scrambling strategy with multi-chess-piece rules, wherein traversal rules and scrambling operations are not predefined; instead, they are dynamically updated according to the real-time state evolution of the 4D-VOFHS. Through comprehensive correlation analysis and differential attack tests, the presented encryption framework achieves outstanding performance metrics: an average NPCR of 99.6%, a UACI of 33.4%, and an average information entropy of 7.9993. Overall, these results verify the strong cryptographic robustness and practical applicability of the scheme, highlighting its great potential for deployment in real-world color image encryption systems.

## 1. Introduction

Conventional cryptographic schemes exhibit limited performance when applied to visual data protection, owing to their inherent structural limitations [1,2]. As emphasized in [3], chaotic dynamical systems possess a series of distinctive characteristics, including extreme sensitivity to initial conditions and system parameters, ergodicity, long-term unpredictability, and pseudorandom behavior. These characteristics lay the theoretical foundation for their cryptographic applications. In the pioneering work by Zhang et al. [4], a multi-image encryption scheme was proposed by integrating chaotic dynamics with DNA computing. Building upon this foundation, subsequent research has focused on the continuous optimization and refinement of image encryption methodologies, integrating chaotic mapping and DNA encoding techniques [5,6,7,8,9]. Notably, image encryption schemes based on memristor-based chaotic systems exhibit significantly higher nonlinear complexity—a critical advantage against modern cryptographic attacks, as verified in [10,11]. Compared with conventional chaotic systems, hyperchaotic systems exhibit higher dynamic complexity and stronger pseudo randomness [12]. A hyperchaotic image encryption algorithm, based on an improved Henon map, was proposed in [13]. Subsequently, Lu et al. [14] constructed a 3D Sine–Henon hyperchaotic system and designed a 3D coordinate matrix to fuse multiple image data into a cubic structure. However, existing integer-order hyperchaotic systems are generally constrained by their fixed integer-order differential structure: the dimensions of system parameters and initial conditions that can serve as keys are relatively limited, and the period chaotic sequences still has room for extension when implemented on digital platforms with finite precision.

Fractional-order chaotic systems exhibit richer and more sophisticated dynamic behviors than conventional integer-order chaotic systems, as reported in [15,16]. Owing to their inherent unpredictability and memory effects, such systems generate chaotic sequences with superior randomness [17]. Qiu et al. [18] proposed an image encryption algorithm based on a hyperchaotic system, which adopts a split random exchange permutation strategy for pixel scrambling and a cross random diffusion scheme for pixel diffusion. This dual mechanism significantly enhances the randomness of pixel position and value variation, thus effectively defending against chosen-plaintext attacks [19]. By designing a memristor-based fractional-order hyperchaotic system and applying it to color image encryption, Qian et al. [20] enhanced the unpredictability of the algorithm via the nonlinearity and memory properties of memristors. For medical images with an arbitrary number of slices, Zhang et al. [21] proposed a novel encryption algorithm that combines 3D spiral transformation and adaptive multi-directional diffusion, achieving efficient and secure encryption using preprocessing strategies and chaotic systems. The synergistic integration of fractional-order hyperchaotic systems with DNA encoding techniques exhibits remarkable performance in image encryption [22]. Ullah et al. [23] constructed S-boxes with high nonlinearity and strong confusion properties by fusing the output sequences of multiple fractional-order chaotic systems, thus effectively enhancing the robustness of the cryptographic algorithm against both differential and linear cryptanalysis attacks. Li et al. [24] designed a fast row–column joint scrambling and diffusion mechanism. However, the aforementioned fractional-order chaotic encryption schemes all adopt a fixed-order design, with the order value remaining constant throughout the encryption process. On the one hand, the order parameter is generally not incorporated into the key set, failing to provide an extra dimension for the key space; on the other hand, a fixed order corresponds to an invariant dynamic structure, which poses potential security risks against parameter identification attacks.

Variable-order fractional chaotic systems introduce time-varying orders, which enhance the ability to disrupt the statistical correlations among image pixels [25,26,27,28]. Based on the conventional fractional-order Lure chaotic system, Priyanka et al. [29] developed a variable-order fractional Lure chaotic system that employs a piecewise function as the order function. Its application to grayscale image encryption effectively improves the security level. However, existing variable-order chaotic encryption schemes mostly adopt functions with simple forms, and the dynamic characteristics brought by time-varying orders have not yet been fully explored.

Additionally, encryption architecture is another critical factor that restricts the overall security and efficiency of image encryption algorithms [30]. The conventional permutation–diffusion architecture usually splits the encryption process into two independent phases, leading to increased computational overhead and time complexity [31,32,33,34]. To address this issue, studies on synchronous encryption have achieved notable progress. As a case in point, Chen et al. [35] proposed a scheme incorporating random permutation and round diffusion based on a playing card game, thereby enhancing robustness through thorough pixel confusion. Yang et al. [36] enhanced encryption performance by realizing synchronous perturbation of pixel positions and values with a fractal matrix and FSM. However, existing synchronous encryption schemes mostly adopt a fixed coupling mode, and game-inspired encryption mechanisms generally only draw on basic game rules. Schemes that deeply integrate game mechanisms with the dynamic characteristics of chaotic systems are still relatively scarce, and the collaborative optimization of security and efficiency still has room for improvement.

The primary contributions are presented as follows:

(1) A novel four-dimensional variable-order fractional hyperchaotic system (4D-VOFHS) with a sinusoidal time-varying order function is proposed. It exhibits richer dynamic behaviors and better initial condition sensitivity than fixed-order counterparts, producing high pseudorandom sequences and effectively expanding the encryption key space.
(2) A chess-gameplay inspired coupled permutation–diffusion cipher model is constructed. Core rules are abstracted into cryptographic operations to realize synchronous encryption, with dynamically adjustable nonlinear diffusion introduced. It outperforms simple game-inspired mechanisms in rule diversity and dynamic adaptability, raising cryptanalysis difficulty.
(3) A synchronous color image encryption algorithm is designed based on 4D-VOFHS and the proposed cipher model. Adopting a block parallel architecture, it introduces inter-chessboard scrambling and neighborhood coupled diffusion, integrating chaotic dynamics and game encryption rules to balance security and computational efficiency.

The rest of this paper is organized as follows: Section 2 provides a detailed elaboration on both the construction mechanism and dynamic properties of the proposed 4D-VOFHS system. Section 3 describes a tightly coupled permutation–diffusion encryption mechanism inspired by the gameplay rules of international chess. Section 4 elaborates on the complete workflow of the proposed encryption scheme. In Section 5, simulation experiments and comprehensive analyses are conducted to validate the effectiveness and practical feasibility of the proposed scheme. Section 6 summarizes the conclusions of this work and outlines future research directions.

## 2. Hyperchaotic System and Analysis

### 2.1. Proposed 4D-VOFHS

Variable-order fractional chaotic systems, whose orders evolve dynamically with time, can accurately reproduce real-world physical systems exhibiting complex dynamic behaviors. Their dynamic properties adapt to temporal variations and environmental changes, leading to simultaneous alterations in their memory characteristics and dynamic responses [37]. Compared with conventional integer-order chaotic systems, they possess superior robustness against external disturbances and parameter uncertainties, thus exhibiting greater application potential in the fields of secure communication and image encryption. The Lorenz chaotic system is a mathematical model consisting of three nonlinear ordinary differential equations. It characterizes the simplified behavior of fluid under thermal convection, specifically a two-dimensional flow model of the atmosphere [38]. On this basis, by coupling the first three systems to construct a high-dimensional nonlinear state function $x_{4}$ and adopting a trigonometric function as the variable-order function, we formulate the new4D variable-order fractional Lorenz system as follows:

(1) $$\{\begin{array}{c} D^{\alpha \left(t\right)}x_{1}=a\left(x_{2}-x_{1}\right)+x_{4}\\ D^{\alpha \left(t\right)}x_{2}=bx_{1}-x_{1}x_{3}-x_{2}\\ D^{\alpha \left(t\right)}x_{3}=x_{1}x_{2}-cx_{3}\\ D^{\alpha \left(t\right)}x_{4}=-dx_{1}x_{3}+ex_{2} \end{array}$$

where a,b,c,d,e denote system parameters. D0Ctα(t) represents the Caputo variable-order derivative:

(2) D0Ctα(t)f(t)=1Γ(1−α(t))∫0t(t−τ)−α(t)f′(τ)dτ (0<α(t)<1),

where the variable-order function is defined as $\alpha \left(t\right)=\beta_{0}+\omega_{0}sin\left(0.1t\right)$.

Consequently, the solution of the variable-order fractional four-dimensional Lorenz system, approximated via the Adomian decomposition method as reported in [39], is constructed as follows:

(3) $${\overset{∼}{X}}_{j}\left(t\right)=\sum_{n=0}^{6}c_{j}^{n}\left(t\right)\cdot \frac{t^{n\alpha \left(t\right)}}{\Gamma \left(n\alpha \left(t\right)+1\right)},\;$$

where$\;{\tilde{X}}_{j}={\left(x_{1j},x_{2j},x_{3j},x_{4j}\right)}^{T}$ stands for the state vector, while$\;C_{j}^{k}\;$denotes the coefficient vector corresponding to the jth state variable in the kth iteration term. The function$\;\alpha \left(t\right)=\beta_{0}+\omega_{0}sin\left(0.1t\right)\;$constitutes a trigonometric-based variable-order law where $\beta_{0}\;$is the base order and $\omega_{0}\;$is the perturbation amplitude. Appendix A provides the complete Adomian decomposition method for the 4D-VOFHS.

**Figure 1:**
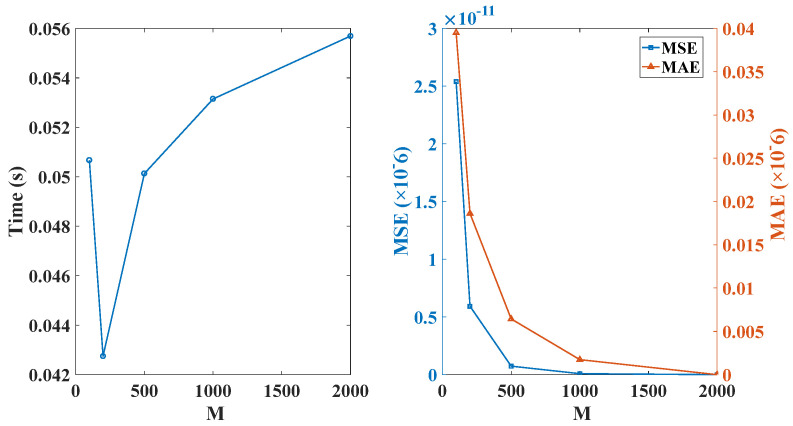
Optimal segment number estimation for the 4D−VOFHS.

To quantitatively analyze the computational complexity of the 4D-VOFHS, the entire iterative process is divided into M segments, where the fractional order within each segment is fixed as a time-averaged constant [40]. This ensures that the long-memory kernels remain identical across all time steps within the same segment, thereby significantly reducing the computational burden. In this study, a sinusoidal variable-order function is defined as α(t)=0.95+0.05sin(0.1t) , and the Adams–Bashforth–Moulton (ABM) predictor–corrector method is adopted as the numerical algorithm with a step size h=0.01 and a total iteration count N = 10,000. Comparative experiments are conducted to evaluate three key metrics: the computational time compared with the same-dimensional, same-type constant fractional chaotic system, as well as the mean squared error (MSE) and the maximum absolute error (MAE) both calculated relative to the 4D-VOFHS. The experimental results are presented in Figure 1.

The experimental results demonstrate that the piecewise constant approximation method effectively accelerates the computation, and a favorable trade-off between computational efficiency and approximation accuracy is achieved when the number of segments is M = 2000. Consequently, the piecewise constant approximation method achieves a good balance between computational efficiency and accuracy. In addition to the conventional system parameters, the form, frequency, amplitude and phase of the order function, as well as the piecewise constant sequence, can all serve as secret keys in the 4D-VOFHS system, which significantly increases the difficulty of brute-force attacks.

### 2.2. Dissipativity Analysis

As demonstrated by Meng et al. [41], the dissipativity property can be verified by deriving the divergence of the vector field of the system. For System (1), the right-hand side function vector is:

(4) $$f=\left[\begin{array}{c} f_{1}\\ f_{2}\\ f_{3}\\ f_{4} \end{array}\right]=\left[\begin{array}{c} a\left(x_{2}-x_{1}\right)+x_{4}\\ bx_{1}-x_{1}x_{3}-x_{2}\\ x_{1}x_{2}-cx_{3}\\ -dx_{1}x_{3}+ex_{2} \end{array}\right].$$

Thus, the divergence is computed as:

(5) $$\nabla \cdot f=\frac{\partial f_{1}}{\partial x_{1}}+\frac{\partial f_{2}}{\partial x_{2}}+\frac{\partial f_{3}}{\partial x_{3}}+\frac{\partial f_{4}}{\partial x_{4}}=-a-1-c.$$

Substitute the parameters into Equation (5):

$$\nabla \cdot f=-a-1-c=-10-1-\frac{8}{3}\approx -13.667.$$

As a result, the system is inherently dissipative; the volume element of all system trajectories contracts exponentially and eventually converges to an attractor.

### 2.3. Stability Analysis

Set$\;D^{\alpha \left(t\right)}x_{i}=0$,

(6) $$\{\begin{array}{c} a\left(x_{2}-x_{1}\right)+x_{4}=0\\ bx_{1}-x_{1}x_{3}-x_{2}=0\\ x_{1}x_{2}-cx_{3}=0\\ -dx_{1}x_{3}+ex_{2}=0 \end{array}$$

The equilibrium points of the system are derived as

$$E_{0}=\left(0,0,0,0\right),\;\;E_{1}=\left(\frac{2}{\sqrt{3}},\frac{112}{3\sqrt{3}},\frac{28}{3},x_{4}^{\left(1\right)}\right),\;E_{2}=\left(-\frac{2}{\sqrt{3}},-\frac{112}{3\sqrt{3}},\frac{28}{3},x_{4}^{\left(2\right)}\right),$$

where the expressions for $x_{4}^{\left(1\right)}$, ${\;x}_{4}^{\left(2\right)}$ are derived from Equation (1).

The Jacobian matrix J is

(7) $$J=(\begin{array}{cccc} -a & a & 0 & 1\\ b-x_{3} & -1 & -x_{1} & 0\\ x_{2} & x_{1} & -c & 0\\ -dx_{3} & e & -dx_{1} & 0 \end{array}).$$

By applying block matrix partitioning to simplify the computation of the Jacobian matrix, we derive the eigenvalues at the equilibrium point $E_{0}=\left(0,0,0,0\right)\;$which are given as $\gamma_{1}\approx 11.893,\gamma_{2}\approx -0.107,\gamma_{3}\approx -22.787,\gamma_{4}=-\frac{8}{3}$. For equilibrium points *E*_1_ and *E*_2_, the corresponding eigenvalues are $\gamma_{1}^{\prime }\approx -13.855,\gamma_{2}^{\prime }\approx -2.667,\gamma_{3,4}^{\prime }\approx 0.094\pm 10.194i$.

As demonstrated by Wei et al. [42], the necessary condition for an equilibrium point to be locally asymptotically stable is that all of its associated eigenvalues satisfy the corresponding stability criterion:

$$\left|arg\left(\lambda_{i}\right)\right|>\frac{\alpha \pi }{2},i=1,2,3,4,$$

where α=α(t)=0.95+0.05sin(0.1t). Since *E*_0_ has positive real eigenvalues λ≈11.893, it does not satisfy the condition clearly. For the equilibrium points $E_{1}$ and $E_{2}$, their corresponding eigenvalues include a pair of complex conjugate eigenvalues whose real parts are positive. Hence, the condition is not satisfied if α is close to 1. Even if the criterion was satisfied for a small α, the system is still unstable due to the positive real parts of the conjugate eigenvalues. Since all equilibrium points are unstable, it is confirmed that the system exhibits chaotic behavior.

### 2.4. Phase Trajectory

**Figure 2:**
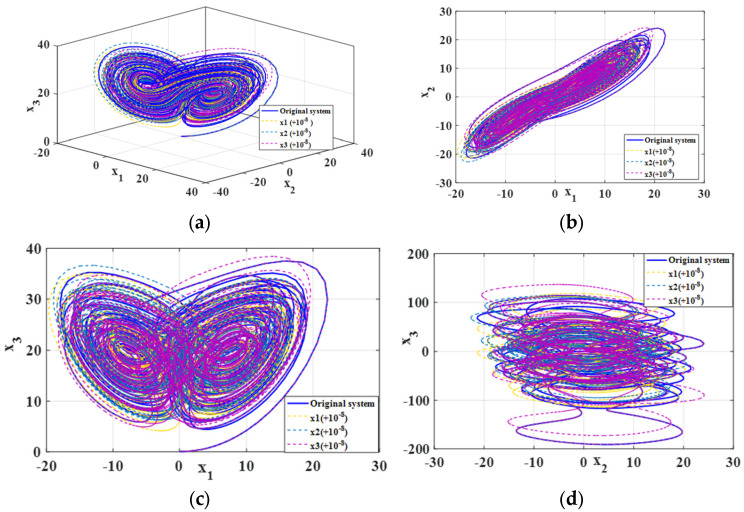
Phase trajectory diagram. (**a**) 3D phase trajectory: (x1, x2, x3); (**b**) 2D phase trajectory: (x1, x2); (**c**) 2D phase trajectory: (x1, x3); (**d**) 2D phase trajectory: (x2, x3).

**Figure 3:**
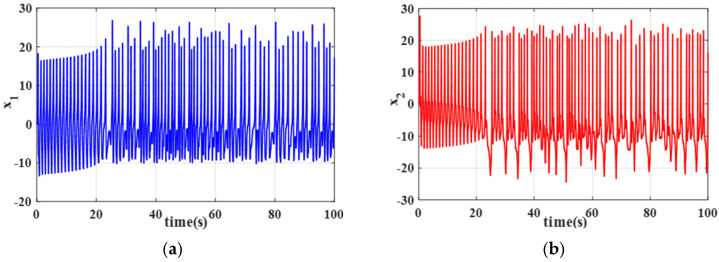

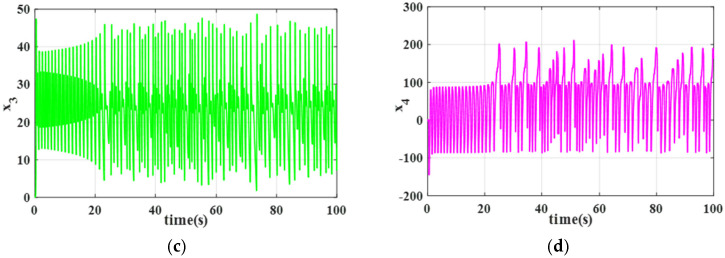
Time history of the state variables. (**a**) Time histories of x1; (**b**) Time histories of x2; (**c**) Time histories of x3; (**d**) Time histories of x4.

As reported in Hamadeh et al. [37], the phase trajectory diagram—a geometric depiction of a dynamic system’s state evolution—comprehensively elucidates the system’s intrinsic by dynamic processes via the trajectories traced in the state space. To characterize the chaotic behavior of the proposed 4D-VOFHS, the system parameters are set to a=10,b=28,c=2.5,d=1.5,e=0.6. The initial conditions are $\left(x_{1}^{0},x_{2}^{0},x_{3}^{0},x_{4}^{0}\right)=\left(0.1,0.1,0.1,0.1\right)$ and the variable order function is α(t)=0.95+0.05sin(0.1t). For the proposed 4D-VOFHS, the projections of its phase trajectories onto separate coordinate planes are illustrated in Figure 2a–d. The analysis of the complex orbital structure demonstrates the existence of complex chaotic characteristics in the system. Additionally, the time-history plots of the four state variables, illustrated in Figure 3, demonstrate aperiodic and irregular oscillatory behavior.

### 2.5. Poincare Map

**Figure 4:**
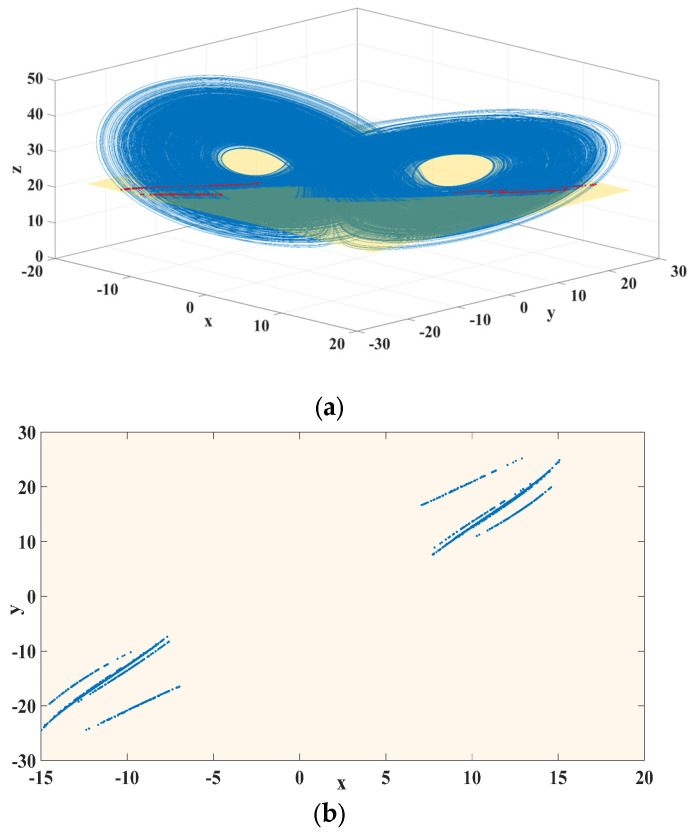
Poincare section and intersection points of the 4D−VOFHS. (**a**) Phase diagram in the x−y−z plane truncated by the z = 0 plane; (**b**) Poincare map.

The Poincare map described by Bagwe et al. [40] is a crucial mathematical tool for analyzing nonlinear dynamic systems which reduces the dimensionality and discretizes the high-dimensional dynamic information of continuous flows into an analyzable discrete map. Figure 4a illustrates the phase space orbits of the system in the x−y−z subspace that intersect the plane z=0, while the corresponding Poincare map is presented in Figure 4b. By visually inspecting these subplots, it can be observed that the points distributed across the Poincare section are densely clustered and randomly scattered, thereby verifying that the system (1) exhibits distinct chaotic behavior. The dark blue shaded area in subfigure b is the plan view of the Peano section.

### 2.6. Sensitivity Analysis

**Figure 5:**
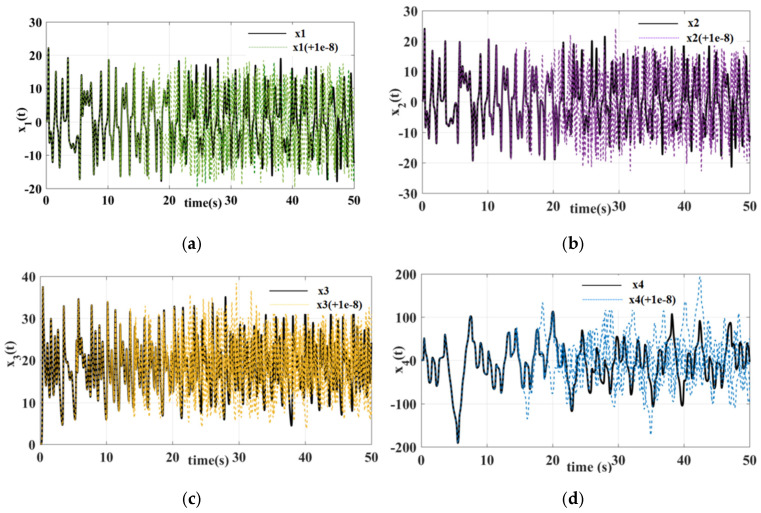
System initial−condition sensitivity. (**a**–**d**) Initial−condition sensitivity analysis of the system.

Sensitivity to initial conditions, as reported by Meng et al. [41], is an intrinsic property that not only governs the dynamic behavior of a system but also determines its suitability for information encryption applications. For an ideal chaotic system, a core defining feature is that even a minuscule perturbation applied to its initial conditions can induce substantial alterations in the system’s dynamic response. To verify the initial condition sensitivity, the initial perturbation is defined as $\left(x_{1}^{0},x_{2}^{0},x_{3}^{0},x_{4}^{0}\right)=\left(0.1+10^{-8},0.1,0.1,0.1\right)$. Figure 5a–d illustrate the evolution of the four state variables under perturbation of $x_{1}$. It can be observed that slight changes in initial conditions result in divergent trajectories over time. This finding confirms that the proposed system exhibits extreme sensitivity to initial conditions, which in turn serves as a crucial underpinning for enhancing the cryptographic resilience of the encryption keys.

### 2.7. Lyapunov Exponent

**Figure 6:**
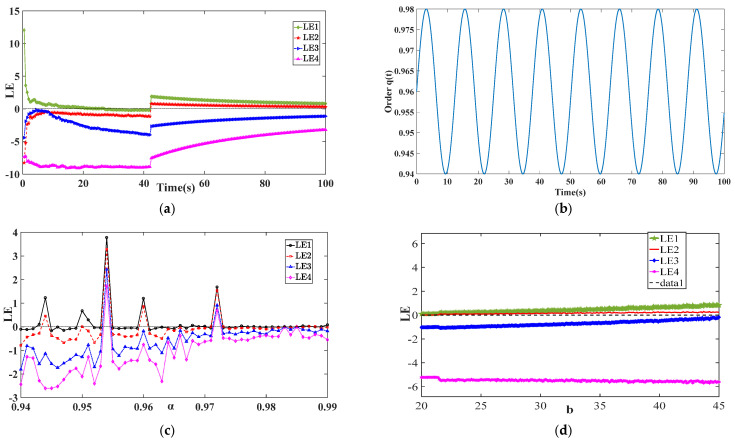
LE behavior analysis. (**a**) Time history of LE; (**b**) Time history of the variable−order function; (**c**) α and LE; (**d**) b and LE.

For the quantitative characterization of the system’s chaotic behavior, the four Lyapunov exponent spectra are derived through time t⩽100 s and a=10,b=28,c=2.5,d=1.5,e=0.6. The initial conditions are $\left(x_{1}^{0},x_{2}^{0},x_{3}^{0},x_{4}^{0}\right)=\left(0.1,0.1,0.1,0.1\right)$. As depicted in Figure 6a, the presence of two positive Lyapunov exponents (LES) LE1=0.97 and LE2=0.25 verifies that the system operates in a hyperchaotic regime. This result substantiates that the system exhibits extreme sensitivity to initial conditions and maintains continuous chaotic dynamic behavior. As depicted in Figure 6c, the system undergoes multiple period-doubling bifurcations with increasing fractional order α. The sharp peaks in the curves correspond to critical bifurcation points, where the system’s dynamic behavior undergoes a phase transition. By analyzing the dynamic variations in the variable-order function, four positive LEs are observed when α=0.955 and when α=0.97 . The maximum Lyapunov exponent reaches its peak value of approximately 3.8, at which point the system exhibits high dynamic complexity and a high orbital divergence rate.

### 2.8. Bifurcation Diagram

**Figure 7:**
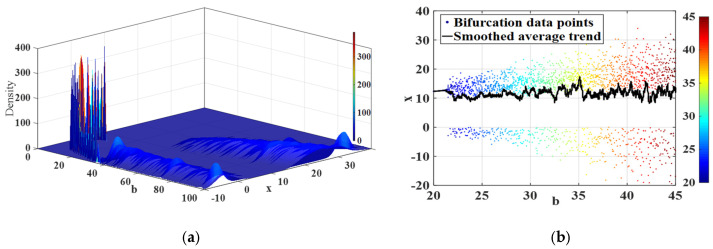
Bifurcation diagram of 4D−VOFHS. (**a**) 3D bifurcation density plot; (**b**) 2D bifurcation.

A bifurcation diagram serves as an indispensable analytical tool for investigating how variations in control parameters affect the long-term dynamic evolution of nonlinear systems, as described by Wei et al. [42]. To visually characterize the system’s transition from periodic to chaotic motion, we sweep a single control parameter and track the corresponding steady-state trajectory points. Numerical simulations are conducted with fixed system parameters a=10,b=28,c=2.5,d=1.5,e=0.6. The initial conditions are.$\left(x_{1}^{0},x_{2}^{0},x_{3}^{0},x_{4}^{0}\right)=\left(0.1,\;0.1,\;0.1,\;0.1\right)$. As illustrated in Figure 7, as the amplitude increases, the system successively undergoes period-doubling bifurcations and orbit splitting, and eventually transitions into a fully chaotic state. This qualitative observation is consistent with and complements the earlier quantitative Lyapunov exponent analysis.

### 2.9. Spectral Entropy

**Figure 8:**
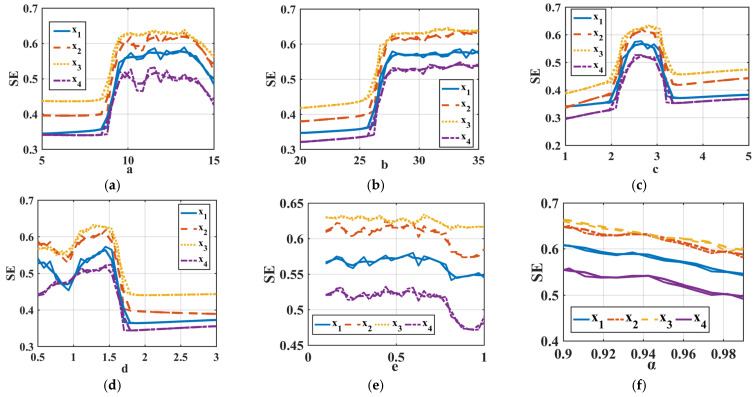
Spectral entropy analysis. (**a**–**e**) SE with control parameters; (**a**–**f**) SE with order parameters α.

**Figure 9:**
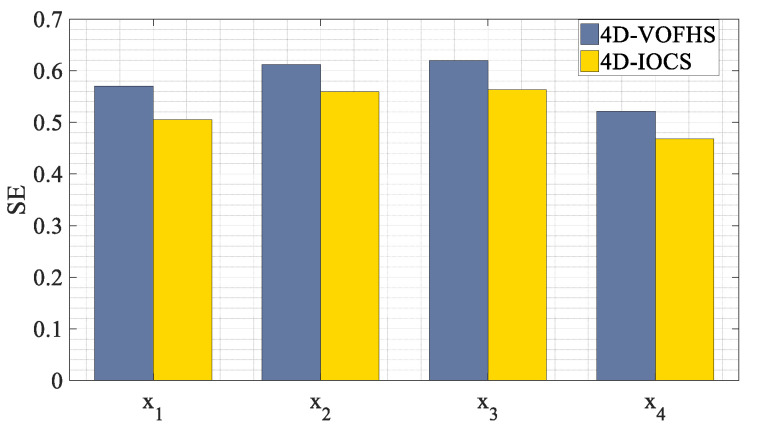
Comparison of SE values for integer-order chaotic systems (IOCSs).

Measures of complexity for fractional-order hyperchaotic systems include permutation entropy, density statistics, the $C_{0}$ algorithm, and spectral entropy (SE), as described by Wang et al. [43]. Among these, spectral entropy is preferred for its high accuracy and low parameter count. By relying on the discrete Fourier transform and the Shannon entropy algorithm, it estimates complexity and reflects the degree of time series disorder in the frequency domain. The parameters are set as a∈[5,15],b=28,c=2.5,d=1.5,e=0.6. The basic order is set to 0.95. As shown in Figure 8a, the variation profile of the corresponding spectral entropy reveals that the system complexity exhibits an initial increase followed by a subsequent decrease as the absolute value of parameter a grows. Setting a=10,b∈[20,35],c=2.5,d=1.5,e=0.6, it can be observed that after a brief period of fluctuation, the complexity stabilizes and exhibits an overall upward trend, as shown in Figure 8b. Figure 8c–e present the relationship between the remaining control parameters and spectral entropy. Similarly, set a=10,b=28,c=2.5,d=1.5,e=0.6, the parameter α∈[0.9,1]. As shown in Figure 8f, the variable-order fractional system possesses more pronounced chaotic characteristics than its integer-order counterpart, which makes it a more suitable candidate for secure information encryption applications. The SE values of the proposed 4D-VOFHS are compared with those of other same-dimensional chaotic systems in Table 1. Figure 9 illustrates its SE comparison results with integer-order chaotic systems of identical type and dimension (i.e., 4D-IOCS).

### 2.10. NIST

As documented in Wang and Teng [43], to rigorously validate the suitability of the pseudorandom sequences generated by the proposed 4D-VOFHS for cryptographic applications, we leverage the National institute of standards and technology (*NIST SP 800-22*) statistical test suite—a universally recognized standard established by NIST—to quantify their statistical randomness. To mitigate the effects arising from the initial transient behavior of the chaotic system, we discard the first 2000 generated data points and convert the remaining sequence into a binary bitstream for subsequent testing. According to the results listed in Table 2, all tests are passed successfully. This confirms that the sequences have satisfactory statistical randomness, which further illustrates that the presented 4D-VOFHS can act as a reliable keystream generator in the field of secure image encryption.

### 2.11. Security Advantages of the Variable-Order Design

**Figure 10:**
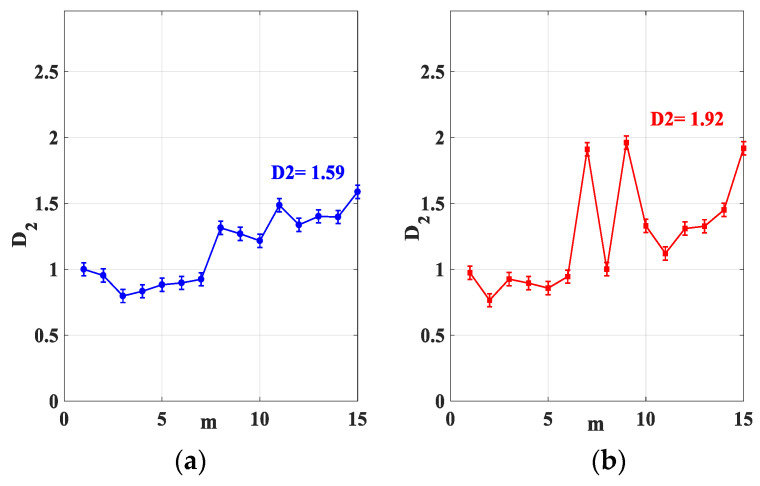
Correlation dimension analysis. (**a**) Correlation dimension of the 4D-COFHS; (**b**) Correlation dimension of the 4D-VOFHS.

To quantitatively verify the superiority of the variable-order design over fixed-order fractional hyperchaotic systems with respect to encryption security, we conduct validation from the following two perspectives [46]. Firstly, the dimensionality of the key space is substantially expanded. For conventional fixed-order fractional hyperchaotic systems, the key set consists solely of system parameters and initial conditions, leading to a restricted key space dimensionality. The proposed variable-order mechanism introduces independent order control parameters for each state variable; each of these parameters exhibits key sensitivity and can be fully integrated into the key space. This dimensionality expansion increases the key space by several orders of magnitude, which far exceeds the standard cryptographic security threshold and drastically increases the computational cost of brute-force attacks. Table 3 compares the key space sizes of the proposed system and conventional fixed-order fractional hyperchaotic systems. Secondly, resistance to phase space reconstruction attacks constitutes the most intrinsic security advantage of variable-order systems over their constant-order fractional hyperchaotic system (4D-COFHS). To quantitatively validate this security gain from dynamic evolution, the Grassberger–Procaccia (GP) algorithm [47] is applied to compute the correlation dimensions of both sequences. As shown in Figure 10, the D_2_ curve of the variable-order system exhibits no saturation across the tested embedding dimension range, with a value of 1.92 at m=15. This non-saturation behavior reflects the higher system complexity arising from its time-varying dynamic properties. According to nonlinear dynamic theory, a higher correlation dimension corresponds to a more intricate fractal geometry in the phase space. Accordingly, the variable-order design intrinsically enhances the algorithm’s robustness against phase-space reconstruction attacks.

## 3. Chess-Gameplay-Inspired Dynamic Mechanism

A chess-gameplay-inspired image encryption algorithm is proposed, which mainly consists of three stages: chessboard reshaping permutation, turn-based attack–defense coupled permutation–diffusion, and inter-chessboard neighborhood diffusion. The designed 4D-VOFHS serves as the primary random sequence generator, with its output chaotic sequences governing the chessboard reshaping rules, move order and attack strategies. This ensures that the encryption process possesses high unpredictability.

### 3.1. Chessboard Reshaping Permutation

During the chessboard reshaping permutation phase, the chessboard layout adjustment is mimicked. Through row and column shifting and inter-channel pixel remixing, the image’s spatial structure and the correlations between channels are effectively eliminated.

Let $I\in Z^{m\times n\times 3}$ represent the original color image. Separate the three RGB channels and concatenate them into a 1D vector. l=3⋅m⋅n denotes the length of the 1D vector obtained by flattering the plain image. Using the random sequence generated by the 4D-VOFHS, the pixel values are permuted to obtain the scrambled 1D vector V(k). In accordance with standard chessboard dimensions, the padded pixel matrix is partitioned into standard 8 × 8 blocks a platform for subsequent chess piece operations.

**Step 1: Calculating the dimensions of the chessboard matrix**

Let the dimensions of the pixel matrix be r×c and let the side length of the matrix after padding be l. Calculations are performed according to Equation (8) to determine whether the padding of original image pixel matrix is needed so that r×c≥l and to ensure the dimensions are multiples of 8.

(8) $$\;\;s=\left\lceil \sqrt{l}\right\rceil ,r=8\cdot \lceil \frac{s}{8}\rceil ,c=8\cdot \lceil \frac{l}{r}\rceil .$$

**Step 2: Padding and reshaping**

If the image pixel matrix requires padding, the padding length g=r×c−l must be calculated. Subsequently, the 4D-VOFHS is used to generate p chaotic values $\left\{x_{l+1},x_{l+2},\ldots ,x_{l+p}\right\}$, which are appended to the initially scrambled 1D pixel vector $V^{\prime }\left(k\right)\;$to obtain the padded 1D pixel vector $V_{1}\left(k\right)$, as show in Equation (9):

(9) $$V_{1}\left(k\right)=\{\begin{array}{cc} V^{\prime }\left(k\right) & 1\leq k\leq l,\\ \lfloor 256\cdot x_{k}\rfloor & l<k\leq l+g. \end{array}$$

where $x_{k}\in \left[0,1\right)$.

Reshape the 1D vector $V_{1}\left(k\right)\;$into a large matrix M(i,j):

(10) $$M(i,j)=V_{1}\left(\left(i-1\right)\cdot c+j\right),i=1,2,\ldots ,r,j=1,2,\ldots ,c.$$

**Step 3: Multi chessboard block mapping**

**Figure 11:**
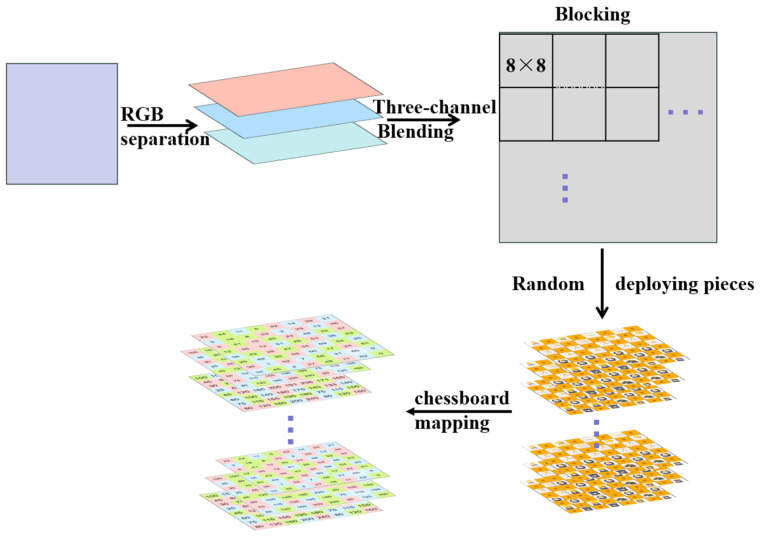
Example of chessboard state matrix mapping.

A bijective mapping is constructed between the padded pixel matrix and the 8 × 8 chessboard matrix employed for chess gameplay. Divide the aforementioned padded large pixel matrix into $k=\frac{\left(r\cdot c\right)}{64}.\;$small pixel matrix chessboards of size 8 × 8. Meanwhile, define the chessboard mapping function $B_{k}:Z_{8}\times Z_{8}\to B$:

(11) $$B_{k}\left(i,j\right)=M\left(8\cdot \lfloor \frac{k-1}{\frac{c}{8}}\rfloor +i,8\cdot \left(\left(k-1\right)mod(\frac{c}{8})\right)+j\right),$$

where i,j∈{1,2,…,8} are the intra-block coordinates. k∈1,2,…,K is the block index. Each chessboard $B_{k}$ corresponds to two state matrices, one of which is the pixel value matrix $P_{k}\in B_{8\times 8}$. The other is the chess piece state matrix $S_{k}\in {\left\{-6,-5,\ldots ,6\right\}}^{8\times 8}$:

(12) $$S_{k}(i,j)=\{\begin{array}{cc} 0 & \mathrm{vacancy}\\ \eta & \mathrm{white}\\ -\eta & \mathrm{black} \end{array},$$

where η=1,2,⋯,6 denotes the type of chess piece. 1 = king, 2 = queen, 3 = rook, 4 = knight, 5 = bishop, and 6 = pawn; this is subsequently used for encryption operations based on chess rules. An example of the chessboard state matrix mapping is given in Figure 11.

**Step 4: Randomly deploying pieces**

In a traditional chess game, there are 16 white pieces and 16 black pieces, which are symmetrically distributed on the upper and lower sides of the board in a fixed pattern, forming a confrontational mode. To expand the cryptographic key space and strengthen the stochastic characteristics of the initial configuration, 64 values $u_{m}=\left\{u_{1},u_{2},\ldots ,u_{64}\right\}$ are extracted from the chaotic sequence. They are sorted in descending order. If equal values occur, ties are broken by index order to obtain $u_{m}^{\prime }$. Take the first 32 positions to place pieces and split them into two groups: the first 16 belong to white, and the remaining 16 belong to black. Distinct from traditional chess placement, the pieces are arranged by sequentially placing the six types, following the order of positions determined by the sorted chaotic values. Piece types are assigned to the 16 positions of each side for each chessboard $B_{k}$ as follows: 1 king (type 1), 1 queen (type 2), 2 rooks (type 3), 2 knights (type 4), 2 bishops (type 5), and 8 pawns (type 6).

### 3.2. Multi-Round Turn-Based Attack Synchronous Encryption

**Figure 12:**
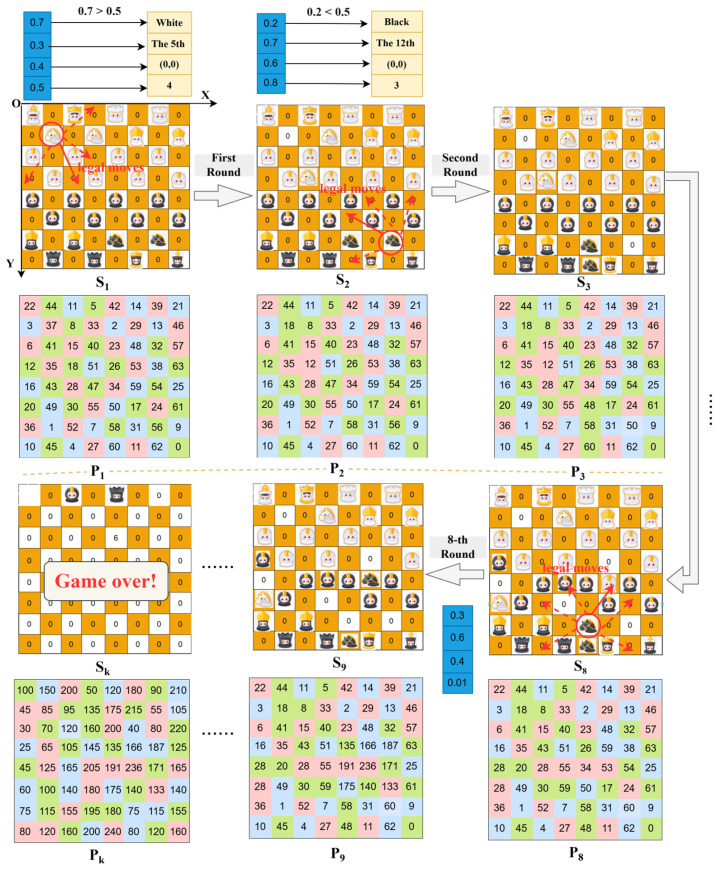
Example visualization of multi-round chess-gameplay synchronous encryption process.

In the turn-based attack–defense phase, there is a closely coupled scrambling-diffusion. A virtual chess match scenario is established with white and black alternately performing moves and captures. The output of each round acts as diffusion feedback for the input to the subsequent round. An example of the multi-round chess-gameplay diffusion process is given in Figure 12 and Algorithm 1. The 4D-VOFHS generates a chaotic sequence ${\left\{x_{1,n},x_{2,n},x_{3,n},x_{4,n}\right\}}_{n=1}^{l^{\prime }}$, where $l^{\prime }=r\times k\times 64\;$to meet the requirements of image encryption. Figure 12 presents an illustrative example of the processing procedure for the multi-round chess-gameplay diffusion process. Set each chessboard $B_{k}$ to undergo h rounds of play for round h=1,2,…,H and chessboard k=1,2,…,K. The detailed operational procedures of the diffusion mechanism are described below.
**Algorithm 1.** Multi-turn-based attack–defense coupled permutation–diffusion.Input: P (8 × 8 pixel block); S (8 × 8 status block); (i_src, j_src): source coordinates; (i_tgt, j_tgt): target coordinates; K_1_, K_2_; λ ∈ [0, 1); s ∈ [1,7]. Output: Updated P, S.1: if S(i_t, j_t) = 0 then 2:     swap (i_src, j_src), (i_tgt, j_tgt) 3:     P(i_tgt, j_tgt) ← P((i_tgt, j_tgt) ⊕ K_1_ + K_2_) mod 256 4:     S(i_tgt, j_tgt) ← S(i_src, j_src); S(i_src, j_src) ← 0 5: else 6:      P(i_tgt, j_tgt) ← f(P(i_src, j_src), P((i_tgt, j_tgt), λ)) % Nonlinear fusion. 7:      for each (δ_i, δ_j) ∈ {−1,0,1}^2^\{(0,0)} 8:             i_n ← ((i_t + δ_i − 1) mod 8) + 1 9:             j_n ← ((j_t + δ_j − 1) mod 8) + 1 10:             P(i_n, j_n) ← P(i_n, j_n) ⊕ CRS(P(i_tgt, j_tgt), s) 11:      end 12:      S(i_tgt, j_tgt) ← S(i_src, j_src); S(i_src, j_src) ← 013: end if

**Step 1: Identification of the initiating player**

This decision is governed by chaotic values generated from the first state variable $x_{1}$ of the 4D-VOFHS, ensuring greater randomness for the starting player of each chessboard in every round. If the sequence value $x_{1,\left(r-1\right)K+k}>0.5$, white moves first; otherwise, black moves first.

**Step 2: Selecting the piece to be moved**

To ensure the unpredictability of the selected chess piece type, random values generated from the chaotic sequence $x_{2}$ are employed. The chessboard state $S_{k}$ is traversed to collect the positions of all surviving pieces belonging to the current player, and the absolute value is taken to select the piece to be moved.

(13) $$P_{Index}=\lfloor x_{2,\left(r-1\right)K+k}\cdot n_{1}\rfloor +1,$$

where *n*_1_ is the number of surviving pieces. This value varies continuously as pieces are captured during the game, thereby adding randomness to the encryption. Let the position of the selected piece be $\left(i_{s},j_{s}\right)$, and its type is $T=\left|S_{k}\left(i_{s},j_{s}\right)\right|$; specifically, 1=King,2=Queen,3=Rook,4=Knight,5=Bishop,6=Pawn.

**Step 3: Calculating the set of legal moves**

Based on piece type T and board state $S_{k}$, calculate the set $M_{1}\;$of legal target positions. Add chaotic perturbation to generate a modified set of legal moves, enhancing security against rule analysis attacks.

(14) $$M_{2}=\left\{\left(\left(i_{t}+\Delta_{i}-1\right)mod8+1,\left(j_{t}+\Delta_{j}-1\right)mod8+1\right)|\left(i_{t},j_{t}\right)\in M_{1}\right\}$$

where $\Delta_{i}=\lfloor 3x_{3,\left(r-1\right)K+k}\rfloor -1,\Delta_{j}=\lfloor 3x_{4,\left(r-1\right)K+k}\rfloor -1$, which are random perturbations determined by the third and fourth chaotic sequence values of the 4D-VOFHS.

**Step 4: Executing the move**

Choose the target $\left(i_{t},j_{t}\right)=M^{\prime }\left[m^{\prime }\right],m^{\prime }=\lfloor x_{\left(r-1\right)K+k+1}\cdot \left|M^{\prime }\right|\rfloor +1$ position from $M_{2}$. The outcome of the move can result in two cases:

(1) If the target position does not contain an opponent’s piece, first swap position $P_{k}\left(i_{s},j_{s}\right)↔P_{k}\left(i_{t},j_{t}\right)\;$ and then perform diffusion processing on the pixel values:
  (15) $$P_{k_{1}}\left(i_{t},j_{t}\right)=\left(P_{k}\left(i_{t},j_{t}\right)⊕K_{1}\right)+K_{2}mod256.$$
  where $K_{1}$ and $K_{2}$ are chaotic sequences generated by the chaotic system.
(2) If an opponent’s piece is present at the target position, execute the capturing process in two stages:
  Firstly, nonlinear fusion is performed,
  (16) $$P_{k_{2}}\left(i_{t},j_{t}\right)=f\left(P_{k}\left(i_{s},j_{s}\right),P_{k}\left(i_{t},j_{t}\right),\lambda \right).$$
  where $f\left(x,y,\lambda \right)=\left[\left(x\cdot y\cdot \lambda \right)mod257\right]⊕\left(\lfloor \frac{x}{2}\rfloor +\lfloor \frac{y}{2}\rfloor \right)mod256$.
  Secondly, this is followed by sputtering diffusion is proposed. The 8 neighboring pixel values are nonlinearly changed through circular right shift and XOR operations:
  (17) $$P_{k_{2}}\left(i_{n},j_{n}\right)=P_{k_{1}}\left(i_{n},j_{n}\right)⊕CRS\left(P_{k_{2}}\left(i_{t},j_{t}\right),s\right).$$
  where $s=\lfloor 7\cdot x_{4,\left(r-1\right)K+k+3}\rfloor +1$ is controlled by the fourth chaotic sequence $x_{4}$ of the 4D-VOFHS. CRS(x,s) represents the 8-bit integer x rotated right by s bits.

**Step 5: Special rule handling for pawn promotion**

In international chess, pawn promotion is an important tactical maneuver. The promotion condition is satisfied when a white pawn reaches the eighth rank or a black pawn reaches the first rank. The likelihood for a pawn to be upgraded into the ith category of chess pieces can be expressed as

(18) $$p_{i}=\frac{e^{x_{j}}}{\sum_{j=1}^{4}e^{x_{j}}},$$

where $x_{j}\left(j=1,2,3,3\right)$ denotes the pseudorandom sequences produced by the adopted 4D-VOFHS model. Each associated pixel value is then converted to

(19) $$P_{k_{3}}\left(i_{t},j_{t}\right)=\left(\lambda_{1}\cdot {\left(P_{k}\left(i_{t},j_{t}\right)\right)}^{2}⊕\lambda_{2}\cdot P_{k}\left(i_{t},j_{t}\right)\right)mod256$$

where $\lambda_{1}$ and $\lambda_{2}$ are parameters derived from the chaotic sequence.

**Figure 13:**
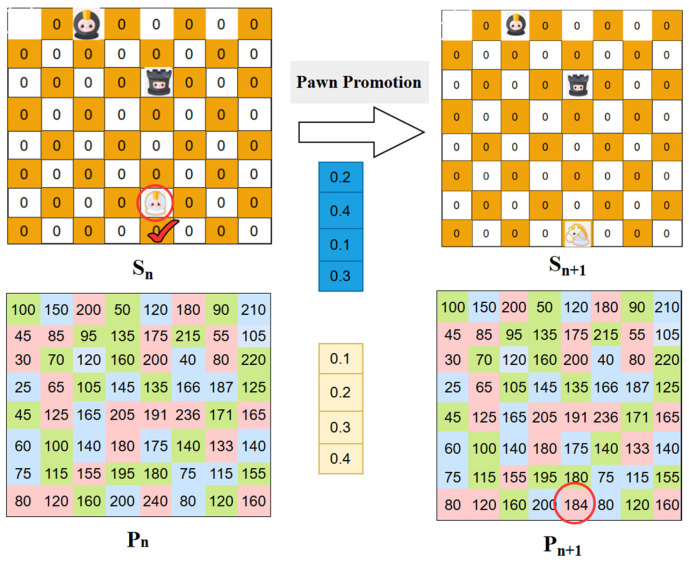
An example of the special rule.

For example, the 4D-VOFHS produces a chaotic sequence $\left(x_{1},x_{2},x_{3},x_{4}\right)=(0.1,0.2,0.3,0.4)$. Calculate the exponent of each value: $e^{x_{1}}\approx 1.105,e^{x_{2}}\approx 1.221,e^{x_{3}}\approx 1.350,e^{x_{4}}\approx 1.491$. The probabilities of a pawn promoting to different piece types are, $p_{1}\approx 0.214,p_{2}\approx 0.236,p_{3}\approx 0.261,p_{4}\approx 0.289$. Another chaotic random number x=0.5 is used to determine the specific piece type. The cumulative probability intervals are [0,0.2) for type 1,[0.2,0.4) for type 2, [0.4,0.7) for type 3 and [0.7,1] for type 4, where type1=Queen, type2=Rook, type3=Knight, type4=Bishop. As it lies in the interval [0.4, 0.7) corresponding to type 3, type 3 (knight) is chosen for the promotion. Given the chaotic sequence $\lambda_{1}=0.01$ and $\lambda_{2}=0.05$, the pixel transformation formula is applied, modifying the pixel value at position (8, 5) to$\;P_{k_{3}}=(\lambda_{1}\cdot {P_{k}}^{2}+\lambda_{2}\cdot P_{k})mod256=184.$ The red checkmark marks the target positions for pawn promotion of white pieces. The detailed process is shown in Figure 13.

**Step 6: Intra-chessboard diffusion**

If there are no opponent pieces on the chessboard, the game ends. Perform diagonal symmetric diffusion on each encrypted chess board:

(20) $$P_{k_{1}}\left(i,j\right)=P_{k}\left(i,j\right)⊕P_{k}\left(j,i\right)⊕P_{k}\left(9-i,9-j\right).$$

### 3.3. Inter-Board Coupled Diffusion

During the inter-chessboard diffusion phase, coupled diffusion across chessboards is conducted upon the conclusion of a round and prior to chessboard restructuring.

**Step 1: Board permutation**

To break the spatial correlation of the chessboard, a random permutation of length K is generated by the 4D-VOFHS. It is used as the scrambling index of the chessboard, denoted as $\pi_{board}=sort\left(K\right)$. Replace the k-th chessboard with the content of the original $\pi_{board}(k)$-th chessboard: $P_{k}^{\prime }=P_{{\pi_{\mathrm{board}}}^{\left(k\right)}}$

**Step 2: Neighborhood diffusion**

**Figure 14:**
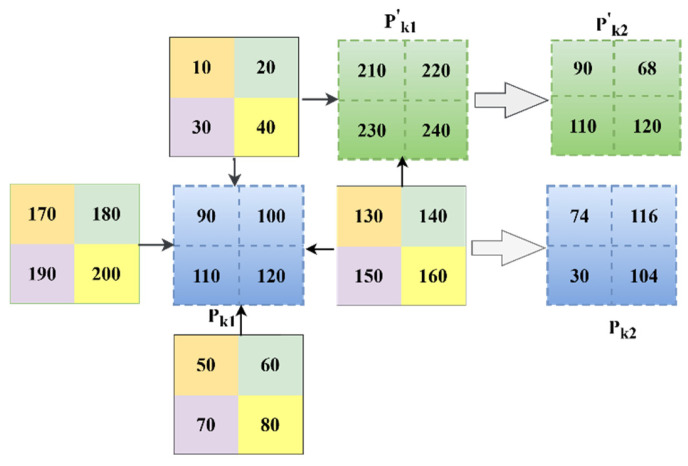
Example visualization of chessboard neighborhood diffusion.

To ensure the pixel values of each chessboard are influenced by neighboring chessboards, thereby enhancing resistance against statistical attacks, neighborhood diffusion is performed:

(21) $$P_{k_{2}}\left(i,j\right)=P_{k_{1}}\left(i,j\right)⊕\underset{l\in N_{4}\left(k\right)}{⨁}P_{l}\left(i,j\right),$$

where $N_{4}\left(k\right)$ represents the adjacency relations (top, bottom, left, and right) of the boards in the matrix. Finally, the chessboard matrices $P_{k_{2}}\left(i,j\right)$ are merged and the three channels are separated to generate the final cipher image *c*. As illustrated in Figure 14, a 4 × 4pixel matrix is used to demonstrate the inter-chessboard neighborhood diffusion mechanism.

## 4. Proposed Encryption Algorithm

**Figure 15:**
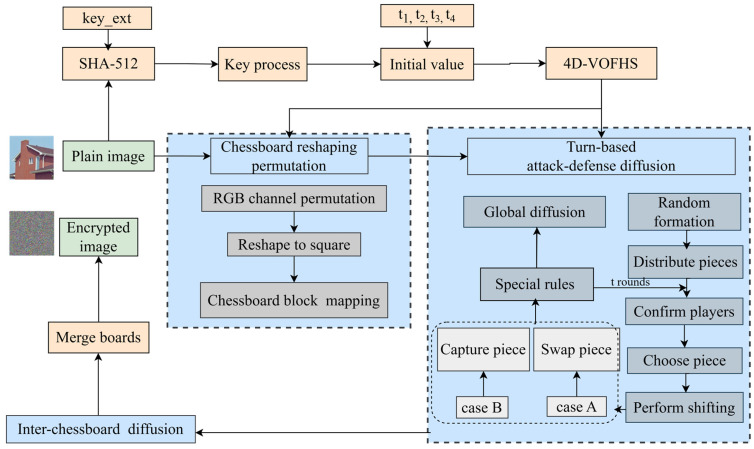
Overall workflow of the proposed color image encryption scheme.

This section introduces a color image encryption framework with synchronous operation, which is designed based on the chess-gameplay mechanism and driven by the 4D-VOFHS. As depicted in Figure 15, the entire workflow consists of key generation, pixel permutation via chessboard reshaping, hybrid permutation–diffusion with turn-based attack–defense interaction, and inter-chessboard diffusion operation.

### 4.1. Key Generation

The key generation process generates the initial conditions and system parameters for the 4D-VOFHS chaotic system. The detailed steps are as follows:

**Step 1: Hash value calculation and segmentation**

The input plain-image I is reshaped into a unidimensional byte array denoted as *vec*(*I*), based on which the corresponding hash digest is computed as $H_{I}=SHA-512\left(vec(I)\right)$. The external secret key $K_{ext}$ is converted into a byte stream to generate its hash value $H_{K}=SHA-512\left(K_{ext}\right)$. These two hash outputs are fused through bitwise XOR to form a 512bit binary hash sequence $H=H_{I}⊕H_{K}$, which is further split into 32 independent segments of 16 bits each.

(22) $$k_{i}=H\left[\left(i-1\right)\times 16:i\times 16\right],i=1,2,\ldots ,32;$$

where $k_{i}$ represents the ith 16-bit binary number.

**Step 2: Intermediate coupling parameters**

To strengthen the nonlinear relationship between subblocks, four intermediate coupling parameters $h_{1},h_{2},h_{3},h_{4}$ are introduced.

(23) $$\{\begin{array}{c} h_{1}=\frac{1}{256}\left[\left(\sum_{j=1}^{8}k_{j}\;\;mod256\right)⊕\left(k_{9}⊕k_{10}⊕\cdots ⊕k_{16}\right)\right]\\ h_{2}=\frac{1}{256}\left[\left(\sum_{j=17}^{24}k_{j}\;\;mod256\right)⊕\left(k_{25}⊕k_{26}⊕\cdots ⊕k_{32}\right)\right]\\ h_{3}=\frac{1}{256}\left(k_{1}⊕k_{2}⊕\cdots ⊕k_{16}\right)\\ h_{4}=\frac{1}{256}\left[\left(\left(\sum_{j=17}^{20}k_{j}+\sum_{j=21}^{24}k_{j}\right)\;\;mod256\right)⊕\left(\left(\sum_{j=25}^{28}k_{j}+\sum_{j=29}^{32}k_{j}\right)\;\;mod256\right)\right] \end{array}$$

**Step 3: Normalization and initial conditions**

By using the intermediate parameters $h_{j}$ and a group of preset perturbation constants $t_{1}=7,t_{2}=13,t_{3}=19,t_{4}=23$, we obtain the initial conditions of the chaotic system through nonlinear mapping.

(24) $$\left\{ \begin{array}{c} x_{1,0}=\frac{\mathrm{mod}((h_{1}\cdot t_{1}+h_{3}\cdot t_{3})\times 10^{4},256)}{\;255}\\ x_{2,0}=\frac{\mathrm{mod}((h_{2}\cdot t_{2}+h_{4}\cdot t_{4})\times 10^{4},256)}{\;255}\\ x_{3,0}=\frac{\mathrm{mod}((h_{1}⊕h_{2}+h_{3}⊕h_{4})\times 10^{4},256)}{\;255},\\ x_{4,0}=\frac{\mathrm{mod}((h_{1}+h_{2}+h_{3}+h_{4})\times 10^{4},256)}{\;255} \end{array}\right.$$

(25) $$\{\begin{array}{c} a=10+\frac{\left(h_{1}⊕h_{3}\right)}{256^{2}}\times 5\\ b=28+\frac{\left(h_{2}⊕h_{4}\right)}{256^{2}}\times 10\\ c=2.5+\frac{\left(h_{1}+h_{2}\right)}{256^{2}}\times 2\\ d=1.5+\frac{h_{3}}{256^{2}}\\ e=0.6+\frac{h_{4}}{256^{2}} \end{array}$$

The variable-order function is designed as $\alpha \left(t\right)=\beta_{0}+\omega_{0}sin(0.1t+\emptyset_{i})$, where $\emptyset_{i}=mod\left(\frac{k_{i}+k_{i+16}}{2^{16}}\times 2\pi ,2\pi \right),i=1,2,3,4.$ $k_{i}$ represents the decimal value of the *ith* 16-bit segment derived in Step 1.

### 4.2. Encryption Process

This subsection introduces a color image cryptosystem that combines the 4D-VOFHS with the chess-gameplay-inspired permutation–diffusion mechanism. A plaintext color image P is transformed into the corresponding ciphertext image C by executing the designed encryption strategy. The entire encryption operation is described in detail in the following content, and the full execution flow is summarized in Algorithm 2 in the form of pseudocode.
**Algorithm 2.** Color image encryption procedure.Input: original image *I*; output: encrypted image *C*.$I_{pad}\leftarrow Padding\left(I,S_{pad}\right)$; $I_{R},I_{G},I_{B}\leftarrow Split\left(I_{pad}\right)$;$H_{I}\leftarrow \mathrm{SHA}-512(\mathrm{vec}(I))$; $H_{K}\leftarrow \mathrm{SHA}-512(K_{ext})$;$H\leftarrow H_{I}⊕H_{K}$; Split H into ${\left\{k_{i}\right\}}_{i=1}^{32}$ where $k_{i}\in \{0,1{\}}^{16}$;$X\leftarrow \mathrm{Iterate}4\mathrm{DVOFHS}(x_{0},L_{total})$;*V*←Concat(SplitChannels(*I*)); l←3mn; $V^{\prime }\leftarrow \mathrm{Scramble}(V,X)$;p←CalcPadSize(l); $V_{pad}\leftarrow \mathrm{PadVector}(V^{\prime },p,X)$; $M\leftarrow \mathrm{Reshape}(V_{pad},r\times c)$;$\left\{B_{k}{\}}_{k=1}^{K}\leftarrow \mathrm{Partition}(M,8\times 8\right)$Deploy Pieces into *S_k_* based on *σ* in order: {King, Queen, Rook, Knight, Bishop, Pawn};Multi-turn-based attack–defense (Algorithm 1);$P_{k}(i,j)\leftarrow P_{k}(i,j)⊕P_{k}(j,i)⊕P_{k}(9-i,9-j)$;End ifReorder Blocks: $P_{k}^{\prime }\leftarrow P_{\pi_{board}(k)}$;$P_{k}^{\prime }(i,j)\leftarrow P_{k}^{\prime }(i,j)⊕{⊕}_{l\in N_{4}(k)}P_{l}^{\prime }(i,j)$;$M_{\mathrm{enc}}\leftarrow$ MergeBlocks({$P_{k}^{\prime }$});$V_{final}\leftarrow \mathrm{Flatten}(M_{enc})$; Remove padding p;$C\leftarrow \mathrm{ReconstructImage}(V_{final},m,n)$;Return *C*;end

**Step 1: Three-channel permutation**

We first extract the red, green and blue components from the input color image, and then convert these three color layers into a 1D vector V, which is then scrambled via a chaotic sequence produced by the 4D-VOFHS to yield $V^{\prime }\left(k\right)$. The 4D-VOFHS is used to generate p chaotic values $\left\{x_{l+1},x_{l+2},\ldots ,x_{l+p}\right\}$, which are appended to $V^{\prime }\left(k\right)$. Then we reshape it into M(i,j).

**Step 2: Chessboard reshaping-based mapping**

M(i,j) is partitioned into k small pixel blocks $B_{k}$, each of size is 8 × 8. Each $B_{k}$ corresponds to two state matrices $P_{k}$ and $S_{k}$, which have a dual mapping relationship.

**Step 3: Random chessboard deployment**

By sorting the random sequence $u_{m}$ from the 4D-VOFHS in ascending order $u_{m}^{\prime }$, position indices are derived to complete the random deployment of pieces in the chessboard matrix $S_{k}$. The chess pieces are placed sequentially in the order of Type  1=King,  Type  2=Queen,  Type  3=Rook,  Type  4=Knight,  Type  5=Bishop, Type  6=Pawn.

**Step 4: Turn-based attack–defense coupled permutation diffusion**

A multi-round attack–defense diffusion mechanism is implemented as described in Section 3.2. 4D-VOFHS controls the playing order, piece types, legal set and path order within the legal set *M*_2_. If the target position is not occupied by an opponent’s piece, the pixels at the two positions are swapped, followed by nonlinear diffusion; otherwise, sputtering diffusion is performed. The special rule of pawn promotion is controlled by $p_{i}$.

**Step 5: Intra-chessboard diagonal diffusion**

When there are no opponent pieces on the chessboard, the current round of the game ends. Subsequently, the pixel matrix $P_{k}\left(i,j\right)$ is diffused based on diagonal symmetric pixels, obtaining $P_{k_{1}}\left(i,j\right)$.

**Step 6: Inter-chessboard permutation and neighborhood diffusion**

$P_{k}^{\prime }$ is scrambling based on the ascending order of a chaotic sequence, followed by neighborhood diffusion between adjacent checkerboards.

**Step 7: Chessboard merging**

The 8 × 8 subblocks $P_{k_{2}}\left(i,j\right)$ are assembled sequentially. Subsequently, the three color components are reconstructed to form the final cipher image c.

### 4.3. Decryption Process

**Figure 16:**
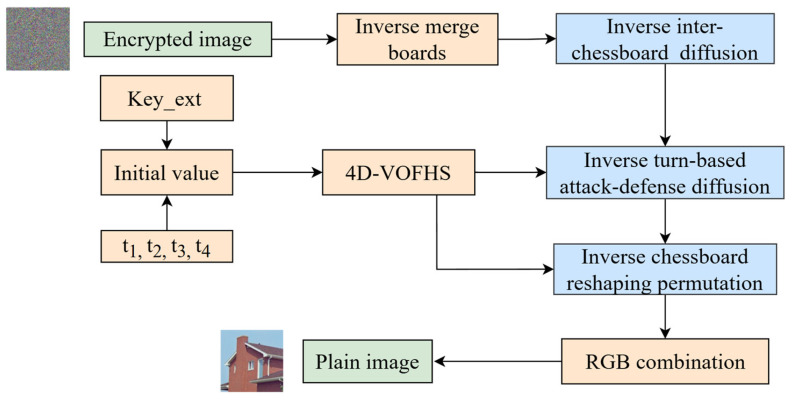
Framework of color image decryption.

The decryption workflow strictly follows the reverse steps of the encryption scheme, where every computational operation is performed in an inverted sequence. For each 8 × 8 encrypted image subblock $B_{k}$, the inverse pixel value transformation is performed based on the corresponding chess piece state matrix $S_{k}$ to reverse the encryption process. Subsequently, padding removal and inverse scrambling operations are conducted, followed by reshaping the result into a color image. The detailed decryption pseudocode is given in Algorithm 3, with its corresponding flowchart depicted in Figure 16.
**Algorithm 3.** The decryption procedure for the color image.**Input:** Encrypted image *C*, $K_{ext}$. **Output:** Plain image *I*;1.$H\leftarrow \mathrm{SHA}-512(\mathrm{vec}(C))⊕\mathrm{SHA}-512(K_{ext})$; $\left\{x_{0},a,b,c,d,e\}\leftarrow \mathrm{InitSystem}(H\right)$;2.$\left\{X\right\}\leftarrow \mathrm{Iterate}4\mathrm{DVOFHS}\left(x_{0},a,b,c,d,e,N_{total}\right);$3.$\left\{C_{R},C_{G},C_{B}\}\leftarrow \mathrm{SplitChannels}(C\right)$;4.For each channel $C_{k}\in \{C_{R},C_{G},C_{B}\}$ do
i.$V_{pad}^{\prime }\leftarrow \mathrm{Flatten}(C_{k})$ii.$V^{\prime }\leftarrow \mathrm{InvScramble}(V_{pad}^{\prime },X)$iii.$M^{\prime }\leftarrow \mathrm{Reshape}(V^{\prime },H_{pad}\times W_{pad})$iv.$\left\{B_{1}^{\prime },B_{2}^{\prime },\ldots ,B_{K}^{\prime }\}\leftarrow \mathrm{Partition}(M^{\prime }\right)$
5.Inverse Round-based Diffusion
(a)For round r=T down to 1 do
i.For each block index k=1 to K do$S_{k}\leftarrow \mathrm{RestoreState}(B_{k}^{\prime },X,r)$$P_{k}\leftarrow \mathrm{InvSplashDiffuse}(B_{k}^{\prime },S_{k},X)$$P_{k}\leftarrow \mathrm{InvNonlinearFuse}(P_{k},X)$;$B_{k}^{\prime }\leftarrow \mathrm{InvMoveTransform}(P_{k},S_{k},X)$ii.End For
(b)End For;
6.For block index k=1 to K doi.For i=1 to 8, j=1 to 8 doii.$B_{k}(i,j)\leftarrow B_{k}^{\prime }(i,j)⊕B_{k}^{\prime }(j,i)⊕B_{k}^{\prime }(9-i,9-j)$iii.End For
End For;7.$\left\{B_{1},\ldots ,B_{K}\}\leftarrow \mathrm{InvPermute}(\{B_{1}^{\prime },\ldots ,B_{K}^{\prime }\},\pi_{board}\right)$(a)For each block index k=1 to K do
i.$NeighborXOR\leftarrow {⨁}_{l\in N_{4}(k)}B_{l}$ii.$B_{k}\leftarrow B_{k}⊕NeighborXOR$
(b)End for;8.$I\leftarrow \mathrm{MergeChannels}(I_{R},I_{G},I_{B})$9.Return I
10.end

## 5. Experiments and Algorithm Analysis

**Figure 17:**
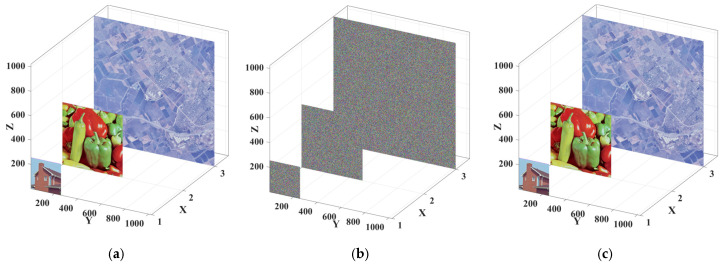
Encryption and decryption results for color images. (**a**) 256 × 256, 512 × 512, 1024 × 1024 plain image P; (**b**) 256 × 256, 512 × 512, 1024 × 1024 cipher image C of P; (**c**) 256 × 256, 512 × 512, 1024 × 1024 decrypted image.

A series of quantitative simulations and systematic security validations are carried out to evaluate the effectiveness of the proposed encryption scheme. All testing tasks are performed on a personal workstation configured with 16 GB DDR4 3200 MHz memory, a 512 GB solid-state drive, and the Windows 10 operating system. All algorithms are implemented in the MATLAB R2022b environment. The benchmark images adopted in the experiments are retrieved from the public USC-SIPI image database (https://sipi.usc.edu/database/ accessed on 1 July 2026), which is widely recognized as a standard dataset for image processing research. Three typical color images, namely House, Peppers, and Stockton, are tested at three mainstream resolutions: 256 × 256, 512 × 512 and 1024 × 1024. The encryption and decryption results of these benchmark samples are displayed in Figure 17a–c. No valid texture or structural details can be identified from the resulting encrypted images, which present a noise-like appearance. The pixel intensities are distributed uniformly, ensuring that the original information of plain images is entirely concealed. Furthermore, the original images can be fully recovered after decryption. No distortion or information degradation occurs, which fully validates the reliability and stability of the proposed encryption scheme.

### 5.1. Histogram

**Figure 18:**
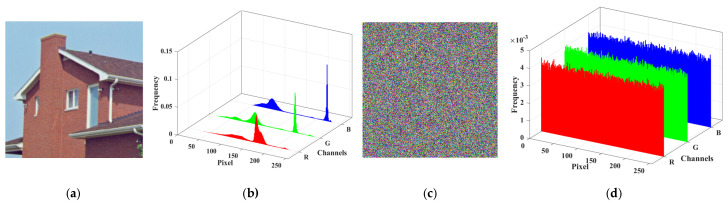

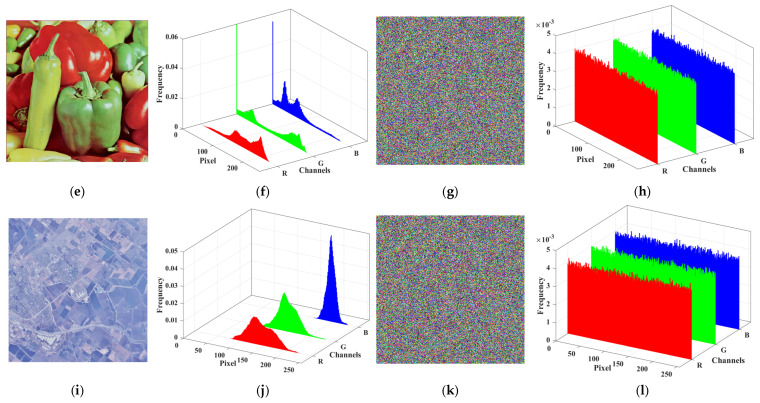
Histogram analysis of test images. (**a**–**c**) Original plain image P; (**d**–**f**) histogram distributions of plain image P; (**g**–**i**) encrypted image C generated from P; (**j**–**l**) histogram distributions of image C.

Histogram analysis serves as an essential metric to examine the performance of image encryption schemes [49]. Figure 18 illustrates the histogram patterns obtained for the three-color test images using our presented encryption method. Visually, the pixel distributions of the encrypted images are flatter and more uniform than those of the original images. Such a balanced statistical characteristic can effectively resist statistical analysis attacks by avoiding the leakage of useful pixel features, which sufficiently validates the excellent security performance of our developed encryption mechanism.

As a quantitative evaluation indicator, the $x^{2}$ metric quantifies the deviation between the pixel intensity distributions of the ciphertext outputs and the theoretically uniform reference distribution. The ideal $x^{2}$ is 293.24783. The $x^{2}$ measurement outcomes obtained by implementing our developed encryption scheme on color visual data are summarized in Table 4. All values fall below the theoretical threshold. This verifies that the proposed algorithm passes statistical tests and serves as an effective encryption approach.

(26) $$\chi^{2}=\sum_{i=0}^{511}\frac{{\left(v_{i}-v_{0}\right)}^{2}}{v_{0}},$$

(27) $$v_{0}=\frac{\left(m\times n\right)}{256}.$$

### 5.2. Correlation Analysis

Adjacent-pixel correlation is a critical factor in evaluating the resistance of image encryption algorithms [50]. For a secure cipher scheme, the strong linear dependence among adjacent pixels in raw images must be eliminated effectively, such that the correlationcoefficients approach zero and the encrypted image exhibit high randomness [51]. The corresponding formulas for quantitative computation are given below.

(28) $$r_{uv}=\frac{cov\;\;\left(u,v\right)}{\sqrt{D\left(u\right)}\sqrt{D\left(v\right)}},$$

(29) $$cov\;\;\left(u,v\right)=\frac{1}{n}\sum_{i=1}^{n}(u_{i}-E\left(u\right))\left(v_{i}-E\left(v\right)\right),$$

(30) $$E\left(u\right)=\frac{1}{n}\sum_{i=1}^{n}u_{i},E\left(v\right)=\frac{1}{n}\sum_{i=1}^{n}v_{i},$$

where u and v denote the gray-level intensities of a pair of adjacent pixels in the image, while E(u) and E(v) represent the mean intensity values of the pixel pairs.

**Figure 19:**
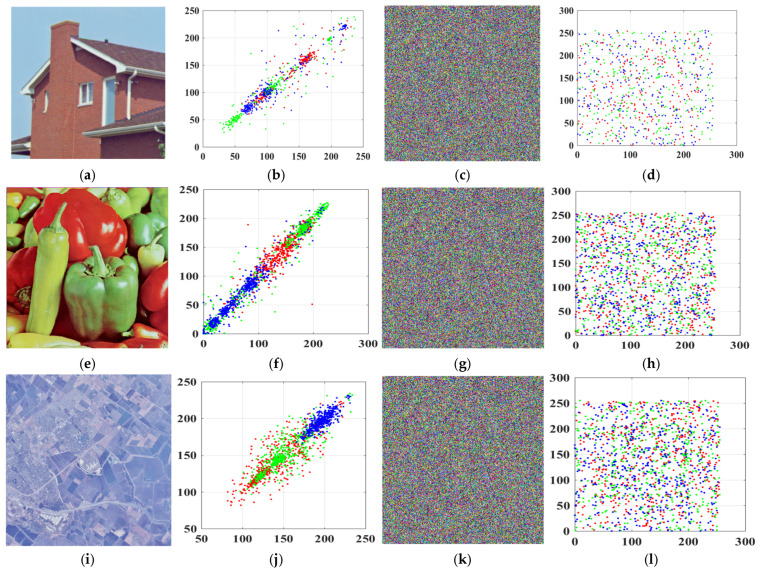
Correlation analysis. (**a**–**c**) Plain images *P*; (**d**–**f**) Correlation of *P*; (**g**–**i**) Cipher images C of *P*; (**j**–**l**) Correlation of *C*.

Three benchmark color images are used in this test: 256 × 256 “House”, 512 × 512 “Peppers”, and 1024 × 1024 “Stockton”. Figure 19 depicts the RGB three-channel correlation distributions for 15,000 randomly chosen adjacent pixel pairs, with statistical computations performed along three typical directions covering horizontal, vertical and diagonal positions for both raw images and their encrypted counterparts, calculated separately for the red, green and blue channels. While adjacent pixels in each channel of the plain images exhibit a strongly clustered distribution along the diagonal, those in the encrypted images show a uniform and random distribution across the entire 2D coordinate space. These experimental results confirm that the proposed encryption scheme achieves an excellent diffusion performance.

As can be observed from the statistical results summarized in Table 5, the correlation coefficients of plaint images in the horizontal, vertical, and diagonal directions range from 0.8 to 0.98, which are very close to the ideal value of 1. This result indicates that adjacent pixels in the original images exhibit strong linear correlation. After encryption, these correlation coefficients decrease to nearly zero in all three directions. A comparative study among our cryptosystem and multiple advanced encryption algorithms on the Peppers test image is summarized in Table 6. The effective elimination of spatial correlation guarantees that the designed encryption strategy satisfies essential security demands for image protection.

### 5.3. Information Entropy

Information entropy, as a conventional performance indicator, is employed to quantify the statistical uniformity of pixel intensity distributions in digital images. For 8 bit grayscale images, the theoretical maximum entropy is 8, as stated in [41]. The entropy results obtained by applying our developed cipher algorithm to the three benchmark test images are summarized in Table 7. As demonstrated by the tabulated data, all computed entropy metrics are very close to the ideal value of 8. This finding confirms that the proposed encryption scheme produces ciphertext images with highly uniform pixel distributions, thus exhibiting strong resistance to statistical analysis attacks.

(31) $$E=-\sum_{i=0}^{l-1}p\left(i\right)log_{2}\;\;p\left(i\right),$$

where the parameter l is the number of gray levels digital image, and p(i) denotes the probability of each gray value in the C encryption image.

### 5.4. MSE and PSNR

As a core quantitative indicator, the peak signal-to-noise ratio (PSNR) is adopted to evaluate the reconstruction quality between the recovered image and its corresponding plaintext image, as reported in Chen et al. [35]. Its mathematical formulation is expressed as follows:

(32) $$PSNR=10log_{10}\;\;\left(\frac{l^{2}}{MSE}\right)$$

(33) $$MSE=\frac{1}{mn}\sum_{i=1}^{m}\sum_{j=1}^{n}(I\left(i,j\right)-K\left(i,j\right){)}^{2}$$

where I(i,j) denotes the pixel intensity of original plain image and K(i,j) represents the pixel values of the corresponding encrypted image. The notation mn specifies the spatial dimensions of the image.

The Structural Similarity Index Measure (SSIM) is designed to quantify the perceptual similarity between two images. The explicit mathematical definition of this metric is given as follows:

(34) $$SSIM\left(I,K\right)=\frac{\left(2\mu_{1}\mu_{2}+C_{1})(2\sigma_{IK+C_{2}}\right)}{\left(\mu_{I}^{2}+\mu_{k}^{2}+C_{1})(\sigma_{I}^{2}+\sigma_{k}^{2}+C_{2}\right)}$$

where $\mu_{I},\mu_{K},\sigma_{I}^{2},\sigma_{K}^{2},\sigma_{IK}\;$ are the mean, variance, and covariance of I,K, $C_{1},C_{2}$. A good encryption algorithm can make SSIM approach 0. Table 8 presents the results.

### 5.5. Differential Attack Resistance

Two widely used quantitative metrics are introduced to validate the resistance against differential attack of the cryptosystem, namely NPCR and UACI. The corresponding experimental results for the proposed scheme are detailed in Table 9. Table 10 presents the comparison of encryption results between the proposed algorithm and other existing algorithms for images of different sizes. In particular, NPCR is used to calculate the percentage of altered pixels in a cipher image. Specifically, the NPCR metric quantifies the proportion of pixel value modifications in the ciphered image, which is induced by altering merely a single pixel within the corresponding original plain image [40]. The explicit mathematical formulation for quantifying the NPCR metric is presented in the following section:

(35) $$NPCR=\frac{\Sigma_{i,j}D\left(i,j\right)}{m\times n}\;\;\times 100\%,$$

(36) $$D\left(i,j\right)=\{\begin{array}{c} 0,C_{1}\left(i,j\right)=C_{2}\left(i,j\right)\\ 1,C_{1}\left(i,j\right)\neq C_{2}\left(i,j\right) \end{array}.$$

The theoretical of NPCR ideal value for 8-bit images is greater than99.6094%.

When a single pixel of the source image is changed, the resulting impact on pixel values in the encrypted image is measured in terms of average intensity by UACI. Its computational formula is provided below:

(37) $$UACI=\frac{1}{mn}\sum_{i=1}^{m}\sum_{j=1}^{n}\frac{\left|C_{1}\left(i,j\right)-C_{2}\left(i,j\right)\right|}{l}\times 100\%$$

### 5.6. Computational Complexity and Efficiency Analysis

The computational complexity of the proposed scheme mainly arises from three core procedures: variable-order chaotic sequence generation, the chessboard dynamic mechanism, and block diffusion. As detailed in Section 2, the piecewise approximation method reduces the time complexity of chaotic sequence generation to O(L). During the dynamic turn-based phase of the chess mechanism, the chaotic sequence is sorted to derive index positions, with a complexity of approximately O(LlogL). Overall, the proposed the encryption algorithm achieves a time complexity of O(LlogL), which scales almost linearly with the total number of pixels. The practical efficiency results are summarized in Table 11. Table 12 evaluates the encryption speed of our designed approach against several state-of-the-art algorithms. The comparative results show that the proposed algorithm outperforms other schemes in terms of execution efficiency.

### 5.7. Key Space Analysis

To resist brute-force attacks, a cryptosystem must have a sufficiently large key space, as stated by Wu et al. [48]. Numerical experiments are conducted using the variable-order fractional Lorenz chaotic system, whose variable-order functions and state variables form the core of the key space. To ensure reliable resistance to brute-force attacks, a cryptographic algorithm must have a key space larger than $2^{100}$, which is a universally acknowledged security standard. The variable-order nature of the proposed algorithm can be regarded as a dynamically order-changing function, which further enhances randomness. The variable-order function is defined as $\alpha (t)=\beta_{0}+\omega_{0}\sin(0.1t)$. The calculation precision is set to $10^{-15}$. The key includes initial values $x_{01},\;x_{02},\;x_{03},\;x_{04}$ and system parameters a,b,c,d,e. The key space size is approximately 2^548^. The key space results and comparative data are presented in Table 13.

### 5.8. Key Sensitivity Analysis

**Figure 20:**
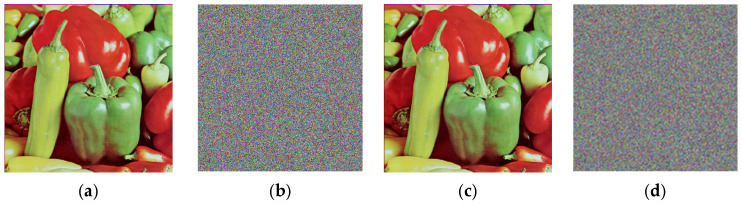
Key sensitivity analysis. (**a**) Plain image; (**b**) Encrypted image; (**c**) Decryption with correct key; (**d**) Decryption with wrong key.

As a core security characteristic, key sensitivity ensures that a slight modification to the decryption key will prevent the correct recovery of the plaintext image from the corresponding ciphertext. To verify this property, a small perturbation is applied to the secret key. The perturbed key is defined as follows:

$$key^{\prime }=\left\{x_{01}+\delta ,x_{02},x_{03},x_{04},a,b,c,d,e,\alpha \left(t\right)\right\}$$

where $\delta =10^{-16}$. The experimental results of attempting to recover the plaintext image using this perturbed key are presented in Figure 20. The plain image is shown in Figure 20a, and the corresponding encrypted result is presented in Figure 20b. The correctly recovered image with the valid key is illustrated in Figure 20c. Figure 20d illustrates the decrypted result when using the slightly perturbed key. These results confirm that the proposed encryption scheme exhibits strong key sensitivity for both encryption and decryption.

### 5.9. Robustness Analysis

**Figure 21:**
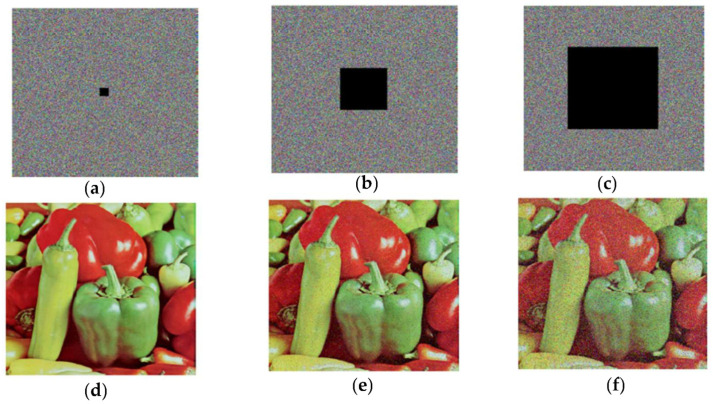
Cropping attack analysis: (**a**–**c**) 5%, 25%, 50% cropped ciphertext images; (**d**–**f**) decrypted images after 5%, 25%, 50% cropping.

**Figure 22:**
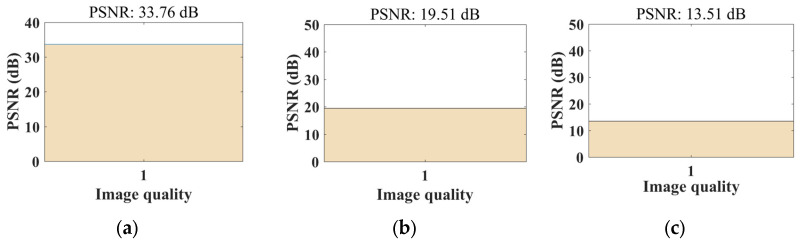
PSNR of cutting attack analysis: (**a**) 5% cropping attack; (**b**) 25% cropping attack; (**c**) 50% cropping attack.

**Figure 23:**
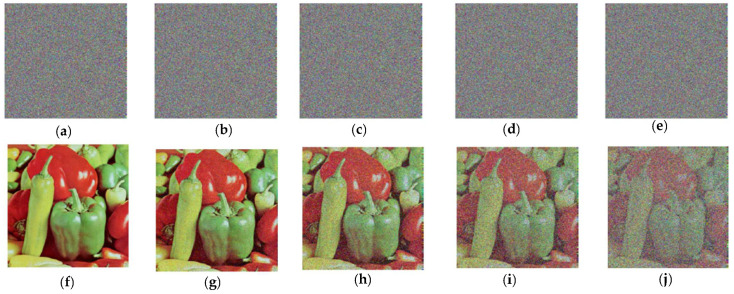
Different noise attack analysis. (**a**–**e**) Noise of 1%, 10%, 30%, 50%, 75%. (**f**–**j**) Corresponding decrypted results under the above noise densities.

During network transmission, ciphertext images may suffer from partial pixel loss and noise contamination, leading to the degradation of pixel information. To evaluate the robustness of an image encryption algorithm, its anti-distortion performance is regarded as a critical metric, as stated by Chen et al. [35]. Figure 21 shows the cropped ciphertext image of the “Peppers” image and the corresponding decrypted image. Figure 22 quantitatively presents the PSNR values of images corrupted under the 5%, 25%, and 50% cropping attacks. Figure 23 illustrates the test results for images corrupted by 1%, 10%, 30%, 50%, and 75% salt-and-pepper noise.

### 5.10. Known-Plaintext and Chosen-Plaintext Attacks Analysis

**Figure 24:**
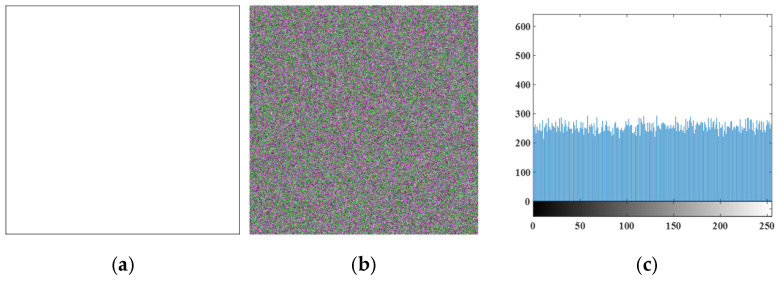

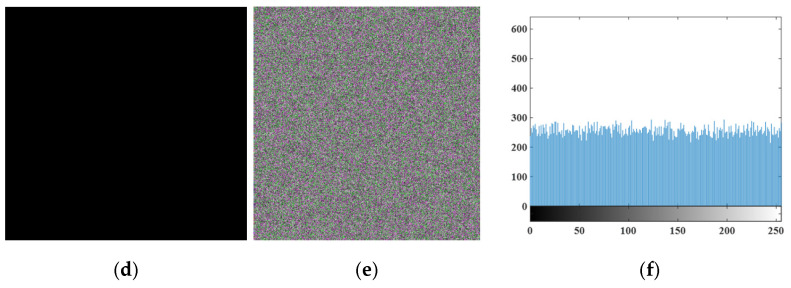
Chosen-plaintext test results for all-white and all-black color images: (**a**) White; (**b**) Cipher image of white C; (**c**) Cipher histogram of white image; (**d**) Black image; (**e**) Cipher image of black image; (**f**) Cipher histogram of black image.

Known-plaintext attack (KPA) and chosen-plaintext attack (CPA) are prevalent modern cryptanalytic approaches, where attackers can infer the encryption key or the underlying system by analyzing pairs of plaintext images and their corresponding ciphertext images [51]. To verify the robustness of the proposed scheme against such attacks, we encrypt two special 256 × 256 images—an all-white image and an all-black image—for testing. The proposed scheme adopts plaintext-dependent encryption, which is highly sensitive to slight changes in the plaintext. Figure 24 visualizes original, all-white and all-black ciphertexts alongside their histograms, and Table 14 lists the corresponding entropy values and correlation coefficients. All ciphertexts exhibit noise-like uniform histograms, with entropy values close to the ideal value of 8 and correlation coefficients near 0, which provides no exploitable plaintext information for attacks. This confirms that the proposed algorithm can effectively resist known-plaintext and chosen-plaintext attacks.

## 6. Conclusions and Outlooks

To address the vulnerability of constant-order chaotic systems to parameter identification attacks and the security-efficiency trade-off in conventional separate permutation–diffusion frameworks, this paper proposes a synchronous color image encryption scheme based on the 4D-VOFHS and a chess-gameplay-inspired dynamic mechanism. Dynamic analysis verifies that the constructed sinusoidal variable-order 4D-VOFHS maintains stable hyperchaotic behavior, with richer dynamic characteristics and a larger key space than constant-order alternatives. The chess-rule-based coupled permutation–diffusion model achieves simultaneous perturbation of pixel positions and values, effectively improving confusion and diffusion performance. Simulation results show that core security metrics including NPCR, UACI and information entropy all reach ideal levels, and the block-parallel design ensures satisfactory computational efficiency.

Despite the above advantages, the numerical solution of variable-order fractional differential equations still involves considerable computational overhead. Future work will optimize the numerical solution algorithm and explore lightweight implementation schemes to reduce computational overhead, further improving the real-time encryption performance of the algorithm on edge terminals and low-power devices and enhancing its practical applicability.

## Figures and Tables

**Table 1:** Comparison maximum SE values for chaotic systems.

Dimension of System	MSE	Reference
4D-IOHS	0.42	[44]
4D-CFHS	0.58	[45]
4D-VOFHS	0.67	Proposed

**Table 2:** NIST statistical test results.

Test Names	*p*-Values				Results
	X_1_	X_2_	X_3_	X_4_	
Frequency Test	0.7577	0.6531	0.3927	0.3261	Passed
Frequency Test within a Block	0.4190	0.2754	0.5336	0.5336	Passed
Cumulative Sums (Forward)	0.4376	0.5673	0.5217	0.4621	Passed
Cumulative Sums (Reverse)	0.6586	0.5832	0.3679	0.5279	Passed
Run Test	0.5673	0.4750	0.6278	0.4961	Passed
Longest Run of Ones in a Block	0.7213	0.6437	0.4102	0.4213	Passed
Binary Matrix Rank	0.5932	0.5932	0.3487	0.3659	Passed
Discrete Fourier Transform	0.2972	0.3606	0.5259	0.6021	Passed
Non-Overlapping Template Matching	0.9369	0.9558	0.9641	0.8912	Passed
Overlapping Template Matching	0.6421	0.6101	0.5739	0.2957	Passed
Maurer’s Universal Matching	0.2491	0.2491	0.4370	0.4751	Passed
Approximate Entropy Test	0.3222	0.3140	0.1529	0.1890	Passed
Serial Test: Serial 1	0.2671	0.2750	0.7943	0.7650	Passed
Serial Test: Serial 2	0.4172	0.5026	0.6970	0.3176	Passed
Linear Complexity	0.4012	0.4687	0.4771	0.5527	Passed

**Table 3:** Comparison of key spaces across different chaotic systems.

System	Control Parameters	Key Space
4D-FOCSC [23]	$\sigma_{1},\;\sigma_{2},\;\sigma_{3},\;\sigma_{4},\;x_{1},\;x_{2},\;x_{3},\;x_{4}$ α, β, γ, k,x,y,z,u,v	$2^{371}$
5D-FOCCS [48]		$2^{498}$
Proposed	$a,\;b,\;c,\;d,\;{e,x}_{1},\;x_{2},\;x_{3},\;x_{4},\beta ,\omega$	$2^{548}$

**Table 4:** Chi-square test.

File Name	Cipher R	Cipher G	Cipher B	Result
House	262.3	284.6	262.1	Passed
Peppers	261.3	256.5	231.4	Passed
Stockton	257.8	238.0	260.6	Passed

**Table 5:** Analysis of RGB horizontal, vertical, and diagonal correlation.

		R		G		B	
Image	Direction	Plain	Cipher	Plain	Cipher	Plain	Cipher
House	Horizontal	0.9951	0.0177	0.9948	0.0079	0.9951	−0.0098
	Vertical	0.9840	0.0449	0.9845	−0.0473	0.9942	−0.0083
	Diagonal	0.9770	−0.0052	0.9798	0.0253	0.9897	−0.0040
Peppers	Horizontal	0.9610	0.0015	0.9826	0.0003	0.9638	−0.0017
	Vertical	0.9612	0.0016	0.9862	−0.0012	0.9686	0.0002
	Diagonal	0.9511	0.00098	0.9751	−0.0022	0.9747	−0.0045
Stockton	Horizontal	0.8153	0.0009	0.7621	0.0001	0.7334	0.0023
	Vertical	0.8056	−0.0001	0.7504	0.0009	0.7214	0.0015
	Diagonal	0.7469	0.0007	0.6896	−0.0006	0.6606	0.0032

**Table 6:** Comparison of RGB horizontal, vertical, and diagonal correlations.

		R	G	B
Method	Direction	Cipher	Cipher	Cipher
Proposed	Horizontal	0.0015	0.0003	−0.0017
	Vertical	0.0016	−0.0012	0.0002
	Diagonal	0.00098	−0.0022	−0.0045
Ref. [39]	Horizontal	0.0032	−0.0014	−0.0013
	Vertical	0.0025	−0.0010	0.0004
	Diagonal	−0.0006	0.0003	0.0007
Ref. [41]	Horizontal Vertical Diagonal	−0.0011 −0.0021 0.0014	0.0007 0.0008 -0.0005	−0.0016 0.0004 -0.0009
Ref. [23]	Diagonal	0.0003	0.0025	−0.0022
	Horizontal	0.0084	0.0061	0.0047
	Vertical	−0.0039	−0.0039	0.0031
Ref. [34]	Diagonal	−0.0013	0.0041	−0.0073
	Horizontal	0.0115	0.0135	0.0133
	Vertical	−0.0194	−0.0146	−0.0134
	Diagonal	−0.0089	0.0094	0.0123

**Table 7:** Information entropy statistics.

	R		G		B	
File Name	Plain	Cipher	Plain	Cipher	Plain	Cipher
House	6.4311	7.9971	6.5389	7.9971	6.2320	7.9990
Peppers	6.3958	7.9994	6.5437	7.9993	6.2161	7.9992
Stockton	6.3988	7.9998	6.5423	7.9998	6.2084	7.9998
Ref. [2]	6.4311	7.9974	6.5389	7.9966	6.2320	7.8976
Ref. [4]	6.3958	7.9993	6.5437	7.9963	6.2161	7.9082
Ref. [6]	6.3988	7.9991	6.5423	7.9959	6.2320	7.8686
Ref. [14]	6.7106	7.9972	6.7962	6.2001	7.9926	7.9921
Ref. [19]	7.3780	7.9916	7.4431	7.9927	7.2334	7.9935

**Table 8:** PSNR and SSIM.

	PSNR	SSIM
File Name	Cipher	Cipher
House	8.1036	0.0081
Peppers	8.1357	0.0063
Stockton	8.1097	0.0089

**Table 9:** NPCR and UACI information.

Image Size	Image	NPCR (%)	UACI (%)
256 × 256	House	99.6175	33.4581
	Clock	99.6208	33.4805
	Moon	99.6154	33.4891
	Barbara	99.6229	33.4416
	Pentagon	99.6136	33.461
512 × 512	Peppers	99.6261	33.5703
	Baboon	99.6182	33.4935
	Lena	99.6109	33.4794
	Mandrill	99.6196	33.4688
	House	99.6273	33.4825
1024 × 1024	Stockton	99.6193	33.4701
	Oakland	99.6274	33.481
	San Diego	99.6241	33.5272
	Los Angeles	99.6187	33.5445
	Bay Bridge	99.6232	33.4978

**Table 10:** NPCR and UACI values of the images under different algorithms.

Image Size	Algorithm	NPCR (%)	UACI (%)
256 × 256	Proposed	99.618	33.4661
	Ref. [34]	99.6312	33.4531
	Ref. [39]	99.6078	33.5708
	Ref. [40]	99.5911	33.4614
512 × 512	Proposed	99.6204	33.4989
	Ref. [34]	99.6162	33.5251
	Ref. [36]	99.608	33.5251
	Ref. [42]	99.5911	33.472
	Ref. [43]	99.6025	33.6038
1024 × 1024	Proposed	99.6204	33.5041
	Ref. [16]	99.6127	33.5031
	Ref. [23]	99.6102	33.4632
	Ref. [32]	99.5278	32.6692

**Table 11:** Performance comparison of encryption efficiency.

Algorithm	Size	Encryption Time (s)
Proposed	256 × 256	0.4402
Ref. [39]	256 × 256	0.3042
Ref. [49]	256 × 256	3.2951
Proposed	512 × 512	1.5137
Ref. [23]	512 × 512	2.7509
Ref. [45]	512 × 512	2.5538
Proposed	1024 × 1024	2.1303
Ref. [48]	1024 × 1024	4.8264
Ref. [49]	1024 × 1024	2.8115

**Table 12:** Comparison of encryption time for 256 × 256 color images.

Algorithm	Permutation	Diffusion	Chaotic Sequence Generation (s)	Total (s)
Ref. [52]	1.0835	2.5863	0.1509	3.8207
Ref. [53]	0.3390	0.8310	0.1508	1.3208
Proposed	0.1176	0.1527	0.1699	0.4402

**Table 13:** Comparison of key space.

Algorithm	Proposed	Ref. [31]	Ref. [10]	Ref. [43]	Ref. [12]	Ref. [32]
Key space	2^548^	2^199^	2^154^	2^200^	2^384^	10^42^

**Table 14:** Entropy and correlation coefficients of plain, cipher images of all white and black.

	Correlation Coefficients			
Image	Horizontal	Vertical	Diagonal	Entropy
All white	0	0	0	0
Cipher of all white	0.0051	−0.0012	0.0086	7.9992
All black	0	0	0	0
Cipher of all black	−0.0028	0.0031	0.0009	7.9994

## Data Availability

The original contributions presented in this study are included in the article. Further inquiries can be directed to the corresponding author.

## Appendix A. Derivation of the 4D-VOFHS via the Adomian Decomposition Method in Section 2.1

The unknown function is decomposed into an infinite series:

(A1) $$x_{i}\left(t\right)=\sum_{n=0}^{\infty }x_{i,n}\left(t\right),$$

with the nonlinear part decomposed by means of Adomian polynomial expansion:

(A2) $$x_{i,0}=c_{i}^{0},\;x_{i,n+1}\left(t\right)=J^{\alpha \left(t\right)}F_{i,n}\left(t\right),\;n\geq 0,$$

where $J^{\alpha (t)}$ is a variable-order integral operator. The equation $F_{i,n}$ can be written as:

(A3) $$\{\begin{array}{l} F_{1,n}=a\left(x_{2,n}-x_{1,n}\right)+x_{4,n}\\ F_{2,n}=bx_{1,n}-x_{2,n}+A_{2,n}\\ F_{3,n}=-cx_{3,n}+A_{3,n}\\ F_{4,n}=ex_{2,n}+A_{4,n} \end{array}$$

where the Adomian polynomials for the nonlinear terms are given by:

(A4) $$\{\begin{array}{l} A_{2,n}=-\sum_{k=0}^{n}x_{1,k}x_{3,n-k}\\ A_{3,n}=\sum_{k=0}^{n}x_{1,k}x_{2,n-k}\\ A_{4,n}=-\sum_{k=0}^{n}x_{1,k}x_{3,n-k} \end{array}$$

The initial term is $c_{j}^{0}=x_{j}\left(0\right)$ and the coefficients of the first termer:

(A5) $$\{\begin{array}{l} c_{1}^{1}=a\left(c_{2}^{0}-c_{1}^{0}\right)+c_{4}^{0}\\ c_{2}^{1}=bc_{1}^{0}-c_{2}^{0}-c_{1}^{0}c_{3}^{0}\\ c_{3}^{1}=c_{1}^{0}c_{2}^{0}-cc_{3}^{0}\\ c_{4}^{1}=ec_{2}^{0}-dc_{1}^{0}c_{3}^{0} \end{array}$$

(A6) $$\{\begin{array}{l} c_{1}^{2}=a\left(c_{2}^{1}-c_{1}^{1}\right)+c_{4}^{1},\\ c_{2}^{2}=bc_{1}^{1}-c_{2}^{1}-\left(c_{1}^{1}c_{3}^{0}+c_{1}^{0}c_{3}^{1}\right),\\ c_{3}^{2}=-cc_{3}^{1}+\left(c_{1}^{1}c_{2}^{0}+c_{1}^{0}c_{2}^{1}\right),\\ c_{4}^{2}=ec_{2}^{1}-d\left(c_{1}^{1}c_{3}^{0}+c_{1}^{0}c_{3}^{1}\right). \end{array}$$

(A7) $$\{\begin{array}{l} c_{1}^{3}=a\left(c_{2}^{2}-c_{1}^{2}\right)+c_{4}^{2}\;\;\;\;\;\;\;\;\;\;\;\;\;\;\;\;\;\;\;\;\;\;\;\;\;\;\;\;\;\;\;\;\;\;\;\;\;\;\;\;\;\;\;\;\;\\ c_{2}^{3}=bc_{1}^{2}-c_{2}^{2}-\left(c_{1}^{2}c_{3}^{0}+c_{1}^{0}c_{3}^{2}\right)-c_{1}^{1}c_{3}^{1}\cdot \frac{\Gamma {\left(\alpha +1\right)}^{2}}{\Gamma \left(2\alpha +1\right)}\\ c_{3}^{3}=-c\;c_{3}^{2}+\left(c_{1}^{2}c_{2}^{0}+c_{1}^{0}c_{2}^{2}\right)+c_{1}^{1}c_{2}^{1}\cdot \frac{\Gamma {\left(\alpha +1\right)}^{2}}{\Gamma \left(2\alpha +1\right)},\\ c_{4}^{3}=ec_{2}^{2}-d\left(c_{1}^{2}c_{3}^{0}+c_{1}^{0}c_{3}^{2}\right)-d\;c_{1}^{1}c_{3}^{1}\cdot \frac{\Gamma {\left(\alpha +1\right)}^{2}}{\Gamma \left(2\alpha +1\right)} \end{array}$$

(A8) $$\{\begin{array}{l} c_{1}^{4}=a\left(c_{2}^{3}-c_{1}^{3}\right)+c_{4}^{3}\;\;\;\;\;\;\;\;\;\;\;\;\;\;\;\;\;\;\;\;\;\;\;\;\;\;\;\;\;\;\;\;\;\;\;\;\;\;\;\;\;\;\;\;\;\;\;\;\;\;\;\;\;\;\;\;\;\;\;\;\;\;\;\;\;\;\;\;\;\;\;\;\;\;\;\;\;\;\;\;\;\\ \;\;c_{2}^{4}=bc_{1}^{3}-c_{2}^{3}-\left(c_{1}^{3}c_{3}^{0}+c_{1}^{0}c_{3}^{3}\right)-\left(c_{1}^{1}c_{3}^{2}+c_{1}^{2}c_{3}^{1}\right)\cdot \frac{\Gamma \left(3\alpha +1\right)}{\Gamma \left(2\alpha +1\right)\Gamma \left(\alpha +1\right)}\\ c_{3}^{4}=-c\;c_{3}^{3}+\left(c_{1}^{3}c_{2}^{0}+c_{1}^{0}c_{2}^{3}\right)+\left(c_{1}^{1}c_{2}^{2}+c_{1}^{2}c_{2}^{1}\right)\cdot \frac{\Gamma \left(3\alpha +1\right)}{\Gamma \left(2\alpha +1\right)\Gamma \left(\alpha +1\right)},\\ c_{4}^{4}=ec_{2}^{3}-d\left(c_{1}^{3}c_{3}^{0}+c_{1}^{0}c_{3}^{3}\right)-d\left(c_{1}^{1}c_{3}^{2}+c_{1}^{2}c_{3}^{1}\right)\cdot \frac{\Gamma \left(3\alpha +1\right)}{\Gamma \left(2\alpha +1\right)\Gamma \left(\alpha +1\right)} \end{array}$$

(A9) $$\{\begin{array}{l} c_{1}^{5}=a\left(c_{2}^{4}-c_{1}^{4}\right)+c_{4}^{4},\;\;\;\;\;\;\;\;\;\;\;\;\;\;\;\;\;\;\;\;\;\;\;\;\;\;\;\;\;\;\;\;\;\;\;\;\;\;\;\;\;\;\;\;\;\;\;\;\;\;\;\;\;\;\;\;\;\;\;\;\;\;\;\;\;\;\;\;\;\;\;\;\;\;\;\;\;\;\;\;\;\;\;\;\;\;\;\;\;\;\;\;\;\;\;\;\;\;\;\;\;\;\;\;\;\;\;\;\;\;\;\\ c_{2}^{5}=bc_{1}^{4}-c_{2}^{4}-\left(c_{1}^{4}c_{3}^{0}+c_{1}^{0}c_{3}^{4}\right)-c_{1}^{2}c_{3}^{2}\cdot \frac{\Gamma \left(4\alpha +1\right)}{\Gamma {\left(2\alpha +1\right)}^{2}}-\left(c_{1}^{1}c_{3}^{3}+c_{1}^{3}c_{3}^{1}\right)\cdot \frac{\Gamma \left(4\alpha +1\right)}{\Gamma \left(3\alpha +1\right)\Gamma \left(\alpha +1\right)},\\ c_{3}^{5}=-cc_{3}^{4}+\left(c_{1}^{4}c_{2}^{0}+c_{1}^{0}c_{2}^{4}\right)+c_{1}^{2}c_{2}^{2}\cdot \frac{\Gamma \left(4\alpha +1\right)}{\Gamma {\left(2\alpha +1\right)}^{2}}+\left(c_{1}^{1}c_{2}^{3}+c_{1}^{3}c_{2}^{1}\right)\cdot \frac{\Gamma \left(4\alpha +1\right)}{\Gamma \left(3\alpha +1\right)\Gamma \left(\alpha +1\right)},\;\;\;\;\;\;\\ c_{4}^{5}=ec_{2}^{4}-d\left(c_{1}^{4}c_{3}^{0}+c_{1}^{0}c_{3}^{4}\right)-dc_{1}^{2}c_{3}^{2}\cdot \frac{\Gamma \left(4\alpha +1\right)}{\Gamma {\left(2\alpha +1\right)}^{2}}-d\left(c_{1}^{1}c_{3}^{3}+c_{1}^{3}c_{3}^{1}\right)\cdot \frac{\Gamma \left(4\alpha +1\right)}{\Gamma \left(3\alpha +1\right)\Gamma \left(\alpha +1\right)}. \end{array}$$

(A10) $$\{\begin{array}{l} c_{1}^{6}=a\left(c_{2}^{5}-c_{1}^{5}\right)+c_{4}^{5},\\ c_{2}^{6}=bc_{1}^{5}-c_{2}^{5}-\left(c_{1}^{5}c_{3}^{0}+c_{1}^{0}c_{3}^{5}\right)-\left(c_{1}^{1}c_{3}^{4}+c_{1}^{4}c_{3}^{1}\right)\frac{\Gamma \left(5\alpha +1\right)}{\Gamma \left(4\alpha +1\right)\Gamma \left(\alpha +1\right)}\\ \;\;\;\;-\left(c_{1}^{2}c_{3}^{3}+c_{1}^{3}c_{3}^{2}\right)\frac{\Gamma \left(5\alpha +1\right)}{\Gamma \left(3\alpha +1\right)\Gamma \left(2\alpha +1\right)},\\ c_{3}^{6}=-cc_{3}^{5}+\left(c_{1}^{5}c_{2}^{0}+c_{1}^{0}c_{2}^{5}\right)+\left(c_{1}^{1}c_{2}^{4}+c_{1}^{4}c_{2}^{1}\right)\frac{\Gamma \left(5\alpha +1\right)}{\Gamma \left(4\alpha +1\right)\Gamma \left(\alpha +1\right)}\\ \;\;\;\;+\left(c_{1}^{2}c_{2}^{3}+c_{1}^{3}c_{2}^{2}\right)\frac{\Gamma \left(5\alpha +1\right)}{\Gamma \left(3\alpha +1\right)\Gamma \left(2\alpha +1\right)},\\ c_{4}^{6}=ec_{2}^{5}-d\left(c_{1}^{5}c_{3}^{0}+c_{1}^{0}c_{3}^{5}\right)-d\left(c_{1}^{1}c_{3}^{4}+c_{1}^{4}c_{3}^{1}\right)\frac{\Gamma \left(5\alpha +1\right)}{\Gamma \left(4\alpha +1\right)\Gamma \left(\alpha +1\right)}\\ \;\;\;\;-d\left(c_{1}^{2}c_{3}^{3}+c_{1}^{3}c_{3}^{2}\right)\frac{\Gamma \left(5\alpha +1\right)}{\Gamma \left(3\alpha +1\right)\Gamma \left(2\alpha +1\right)}. \end{array}$$
